# Cell type and cell signalling innovations underlying mammalian pregnancy

**DOI:** 10.1038/s41559-025-02748-x

**Published:** 2025-07-01

**Authors:** Daniel J. Stadtmauer, Silvia Basanta, Jamie D. Maziarz, Alison G. Cole, Gülay Dagdas, Gilbecca Rae Smith, Frank van Breukelen, Mihaela Pavličev, Günter P. Wagner

**Affiliations:** 1 Department of Ecology & Evolutionary Biology, Yale University, New Haven, CT USA; 2 Konrad Lorenz Institute for Evolution and Cognition, Klosterneuburg, Austria; 3 Department of Evolutionary Biology, University of Vienna, Vienna, Austria; 4 Department of Neuroscience and Developmental Biology, University of Vienna, Vienna, Austria; 5 School of Life Sciences, University of Nevada, Las Vegas, Las Vegas, NV USA; 6 Complexity Science Hub Vienna, Vienna, Austria; 7 Department of Animal Science, Texas A&M University, College Station, TX USA

**Keywords:** Evolutionary developmental biology, Coevolution, Comparative genomics

## Abstract

How fetal and maternal cell types have co-evolved to enable mammalian placentation poses a unique evolutionary puzzle. Here we integrate and compare single-cell transcriptomes from six species bracketing therian mammal diversity: opossum (a marsupial), Malagasy common tenrec (an afrotherian), mouse and guinea pig (rodents), and macaque and human (primates). We identify a conserved transcriptomic signature of invasive trophoblast across eutherians, probably representing a cell type family that radiated with the evolution of haemochorial placentation. In the maternal stroma, comparative analysis reveals that the endocrine decidual cell evolved from an immunomodulatory predecidual cell type retained in *Tenrec* and resembling early human decidua. Fetal and maternal cell signalling shows a pronounced tendency towards disambiguation—the exclusive expression of ligands by only one partner—although few ligand–receptor pairs follow an escalatory arms race dynamic. Finally, we reconstruct the uteroplacental cell–cell communication networks of extinct mammalian ancestors, identifying signalling innovations and widespread integration of fetal trophoblast and maternal decidual cells into signalling networks. Together, these results reveal a dynamic history of cell type innovation and co-evolution at the fetal–maternal interface.

## Abstract (web summary)

Single-cell transcriptomes from the fetal–maternal interface of six species of mammals reveal a conserved gene expression signature of invasive trophoblast, stepwise evolution of decidual stromal cell types and co-evolutionary patterns in cell–cell signalling.

## Main

The origin of new tissues and organs underlies the evolution of multicellular complexity. How higher-level innovations emerge from changes to cell types and their signalling interactions remains unclear. One of the most intense sites of cell signalling in the body is the fetal–maternal interface, where fetal cells from the placenta invade and establish communication with maternal endometrium. Invasive (haemochorial) placentation and the corresponding uterine transformation known as decidualization first evolved in placental (eutherian) mammals^1,2^. Here, we trace the fetal–maternal interface to its evolutionary origin and ask: how does a complex novelty, composed of interdependent parts, come to be assembled in evolution?

**Fig. 1:**
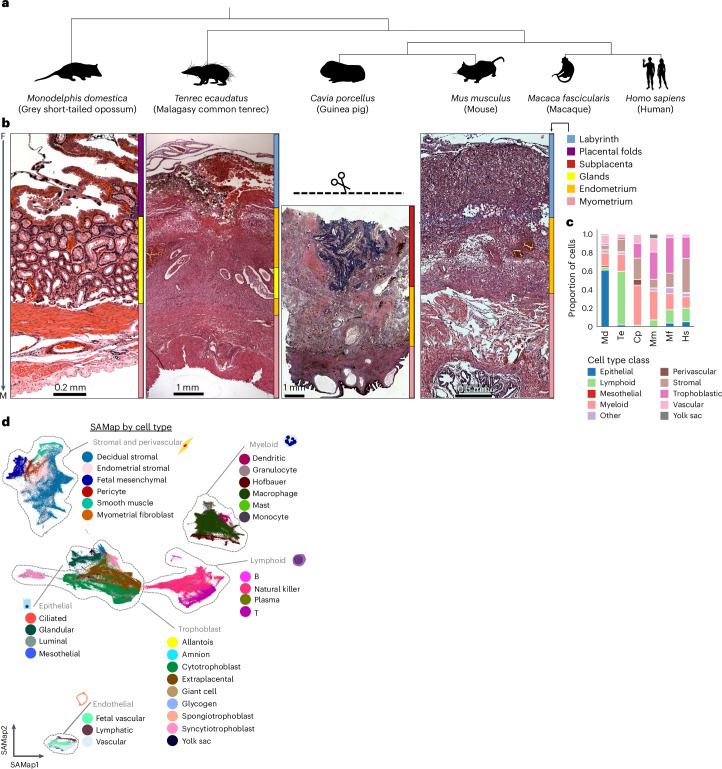
Single-cell transcriptomic atlases of six mammalian species spanning the diversification of viviparity. **a**, Cladogram showing evolutionary relationships between the species sampled. **b**, Histology of the fetal–maternal interface of *M. domestica*, *T. ecaudatus*, *C. porcellus* and *M. musculus*. Colour bars reflect tissue regions (legend on right) from fetal (F) to maternal (M). **c**, Proportional abundances of cell types belonging to major cell type classes in each sample. Cp, *C. porcellus*; Hs, *H. sapiens*; Md, *M. domestica*; Mf, *M. fascicularis*; Mm, *M. musculus*; Te, *T. ecaudatus*. **d**, Unified cross-species transcriptomic embedding (SAMap), coloured by cell type, with prominent divisions annotated in grey. Silhouettes from PhyloPic under a Creative Commons licence: human, NASA (PDM 1.0); tenrec, Yan Wong (CC0 1.0); guinea pig, D.S. (CC0 1.0); opossum, D.S. (CC0 1.0); macaque, Jane Whitehouse (CC0 1.0); mouse, Michael Keesey (PDM 1.0).

Answering this question demands a comparative approach. Single-cell atlases of the human fetal–maternal interface have mapped cell–cell interaction networks governing this chimeric organ^3–5^. Yet, the placenta is among the most rapidly evolving mammalian traits. We present single-cell data from the mid-gestation fetal–maternal interface of six species spanning key nodes in mammal phylogeny (Fig. 1a): the grey short-tailed opossum (*Monodelphis domestica*), a non-deciduate marsupial, the Malagasy common tenrec (*Tenrec ecaudatus*), an afrotherian with ‘primitive’ reproductive traits^6,7^ and four Euarchontoglires—the guinea pig (*Cavia porcellus*), mouse (*Mus musculus*), macaque (*Macaca fascicularis*)^8^ and human (*Homo sapiens*)^4,5^.

We map homologous cell types across species, including trophoblast populations in guinea pig and tenrec that share a gene expression signature with primate extravillous trophoblast. We discover a predecidual cell type in *Tenrec* that suggests a first stage in the evolution of the decidual stromal cell. Reconstructing ancestral cell–cell signalling networks, we show how novel cell types became functionally integrated into the fetal–maternal interface. Finally, we test two long-standing predictions: that fetal and maternal cell signalling repertoires evolve towards disambiguation^9^; and that co-evolution drives signalling escalation between fetal ligands and maternal receptors^10^. In so doing, we chart the evolutionary history of cell types and signalling across the origin and diversification of mammalian placentation.

## Results

### Therians differ in placental and uterine cell types

**Extended Data Fig. 1:**
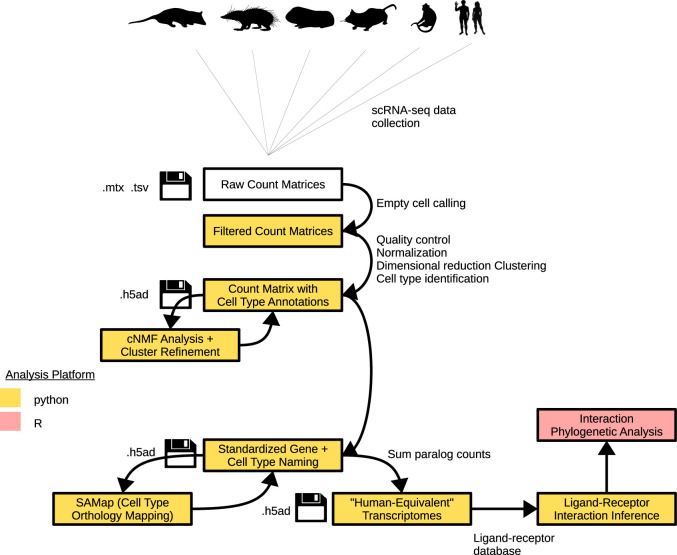
Flow chart of analytical design. After genome alignment, feature-cell barcode matrices were analyzed to annotate cell types on an individual-species basis, followed by cross-species analysis and ligand-receptor interaction inference. Silhouettes from PhyloPic under a Creative Commons licence: human, NASA (PDM 1.0); tenrec, Yan Wong (CC0 1.0); macaque, Jane Whitehouse (CC0 1.0); mouse, Michael Keesey (PDM 1.0).

**Extended Data Fig. 2:**
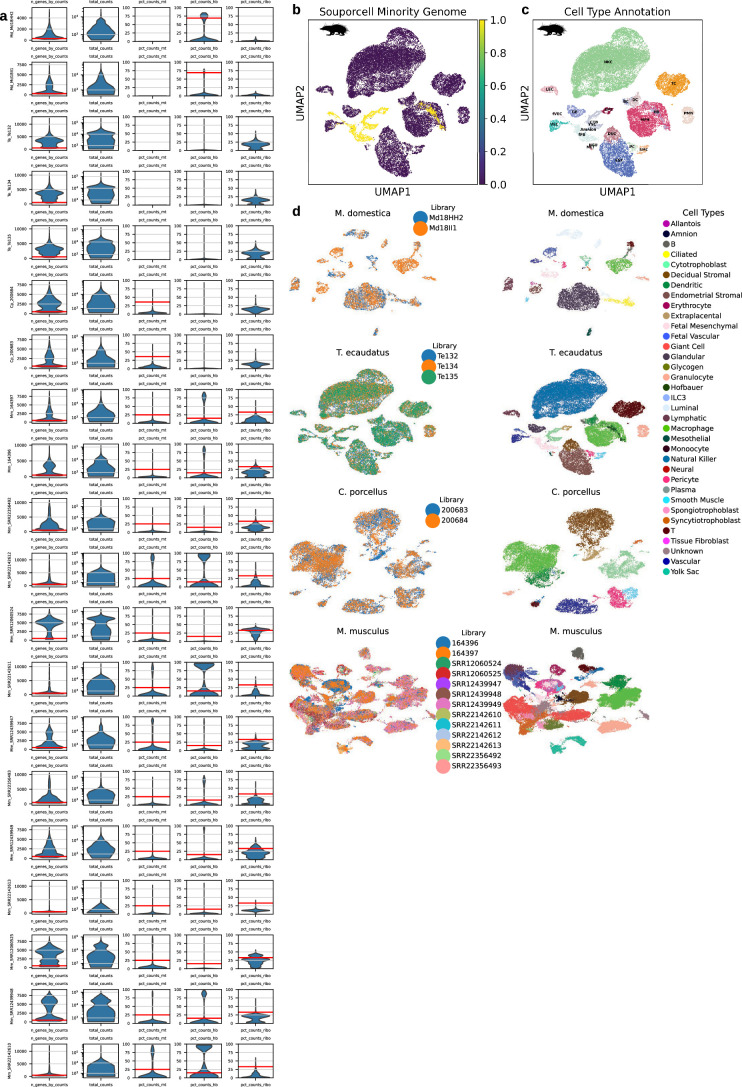
Single cell RNA-seq quality control. **a**. Violin plots of quality control metrics for all libraries of species for which quality control was applied (excluding human and macaque which were quality-controlled by the original authors). n_genes_by_counts: number of unique genes captured per cell; total_counts: total unique mRNA per cell; pct_counts_mt: percent of mitochondrial counts; pct_counts_hb: percent of hemoglobin gene counts; pct_counts_ribo: percent of ribosomal gene counts. Red lines correspond to filtration thresholds. **b**. Souporcell calls for Tenrec ecaudatus (k = 2 genomes) assigned to the less frequent genome (assumed fetal). **c**. UMAP embeddings of Tenrec cells colored by cell subtype. **d**. Species-specific UMAP embeddings colored by library of origin (left) and annotated cell type (right) for the four species with de novo annotations. Source data

**Extended Data Fig. 3:**
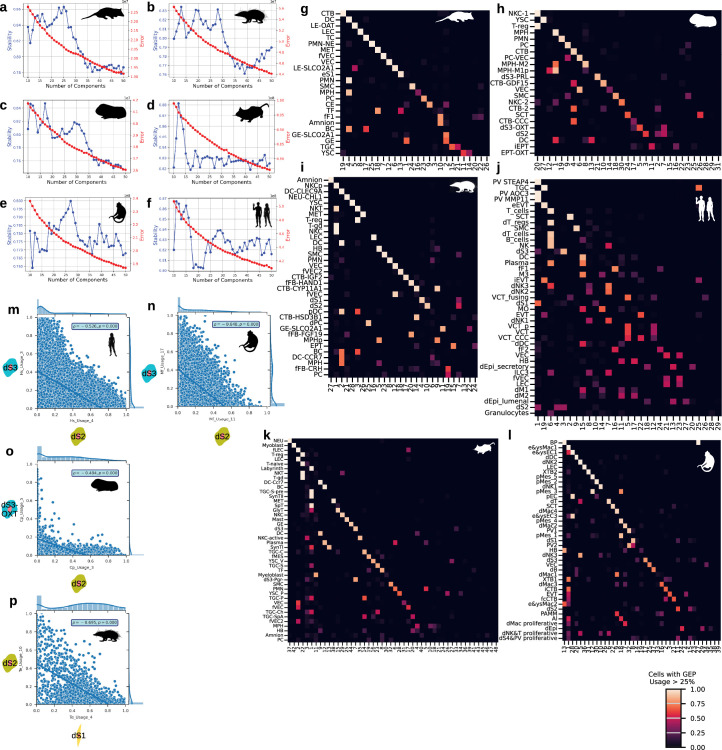
NMF analysis. **a**-**f**. Optimal numbers of gene expression programs (NMF factors) were determined by identifying local stability values preceding a sudden drop. Monodelphis domestica: K = 26; Tenrec ecaudatus: K = 28; Cavia porcellus: K = 31; Mus musculus: K = 48. Homo sapiens: K = 29. Macaca fascicularis: K = 39. **g**-**l**. Confusion matrices showing the mean activity score (ranging from 0 to 1) of cells in each cluster, taken from all cells with scores greater than 0.25. **m**-**p**. Representative “Type II” and “Type III” decidual gene expression program activity scores across individual stromal cells of human (**m**), macaque (**n**), and guinea pig (**o**) show a diversity from exclusively Type II or Type III to a mixture of both programs. An equivalent comparison of Type I and II in the tenrec is depicted in (**p**). Silhouettes from PhyloPic under a Creative Commons licence: human, NASA (PDM 1.0); tenrec, Yan Wong (CC0 1.0); macaque, Jane Whitehouse (CC0 1.0); mouse, Michael Keesey (PDM 1.0).

We assembled single-cell transcriptomic atlases from the uteroplacental interface of six mammal species spanning key points in placental evolution (Fig. 1b). Samples were collected after placental development but before the onset of labour. Data processing followed a standardized workflow, including dead cell and doublet removal, dimensional reduction, Leiden clustering, and annotation refinement informed by cross-species mapping (Extended Data Fig. 1 and Methods). The curated cell atlas includes 599,674 transcriptomes (454,521 whole-cell and 145,153 nuclear-only), with 66,057 unique to this study and an average of 10,617 unique transcripts per cell (Extended Data Fig. 2). Cell clusters were annotated using marker gene expression and histology. To optimize clustering resolution, we applied non-negative matrix factorization (NMF)^11^ to identify co-regulated gene modules (Extended Data Fig. 3), merging clusters lacking distinct NMF modules or markers. Detailed cell type descriptions are provided in Supplementary Table 1.

**Extended Data Fig. 4:**
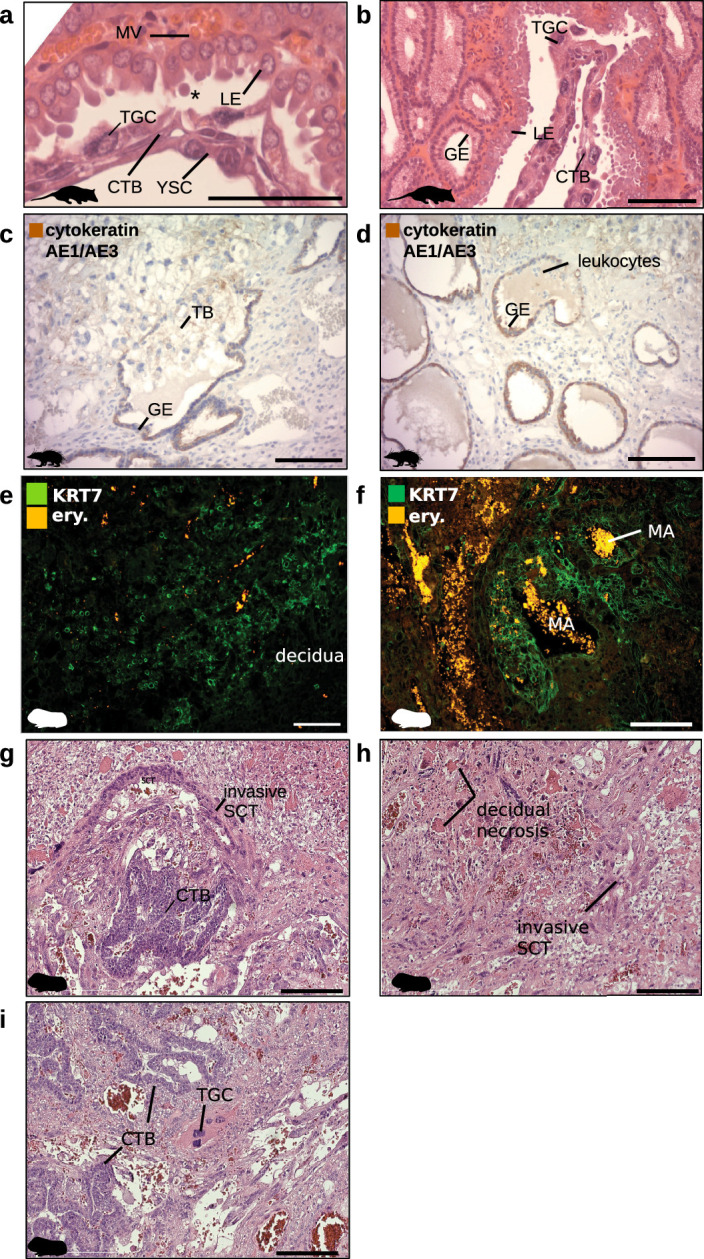
Additional histological identification of placental cell types. **a**-**b**. The opossum fetal-maternal interface, showing 3 layers of yolk sac endoderm (YSC), cytotrophoblast (CTB), and syncytial knots (TGC), in apposition with maternal luminal epithelium (LE) which produces secretory bodies (*); maternal glandular epithelium (GE) lies closely underneath the lumen. **c**-**d**. The tenrec invasion front, where trophoblast invades into mucus-filled glands, eroding the glandular epithelium (GE) immunoreactive for pan-cytokeratin stain (CK AE1/AE3). **e**-**f**. KRT7+ trophoblast in the guinea pig surrounding decidual stroma (left) and maternal arteries (MA; right). **g**-**h**. Invasive syncytiotrophoblast (SCT) in the guinea pig emanates from subplacental cytotrophoblast (left) and extends into stroma where necrotic patches develop (right). **i**. Trophoblast giant cell observed in the subplacenta of the guinea pig (proximate to subplacental CTB), but these cells were not captured by droplet-based sequencing. Scale bars: 200 μm. Silhouettes from PhyloPic under a Creative Commons licence: tenrec, Yan Wong (CC0 1.0); macaque, Jane Whitehouse (CC0 1.0); mouse, Michael Keesey (PDM 1.0).

Tissue organization (Fig. 1b) and cell composition (Fig. 1c) varies across species. Eutherian placentas establish two structures that may be called a fetal–maternal interface: a vascular interface, for circulatory exchange, and a cellular interface, where invading trophoblast contacts maternal decidua. The latter is the focus of our analysis owing to its potential for co-evolved cell–cell signalling. In the tenrec, guinea pig and mouse, the vascular interface takes the form of a trophoblast labyrinth surrounding maternal blood, whereas the primate vascular interface consists of fetal villi reaching into a maternal blood sinus (Fig. 1b). The single interface of the opossum, a marsupial with limited placental invasion, consists of a folded trophoblast matrix coating a maternal epithelium overlying a capillary network^12^ (Extended Data Fig. 4a). Uterine glands, which provide histotrophic nutrition, are expansive (Fig. 1b), reflected in the abundance of epithelial cells captured (>50%; Fig. 1c). Histotrophic glands are also present in the tenrec (Extended Data Fig. 4b), whereas in rodents and primates histotrophy ceases before the completion of placentation. All five eutherian species had greater proportions of stromal cells than the opossum (8%; Fig. 1c), reflecting a derived role of the decidual stroma in forming the eutherian fetal–maternal interface.

**Extended Data Fig. 5:**
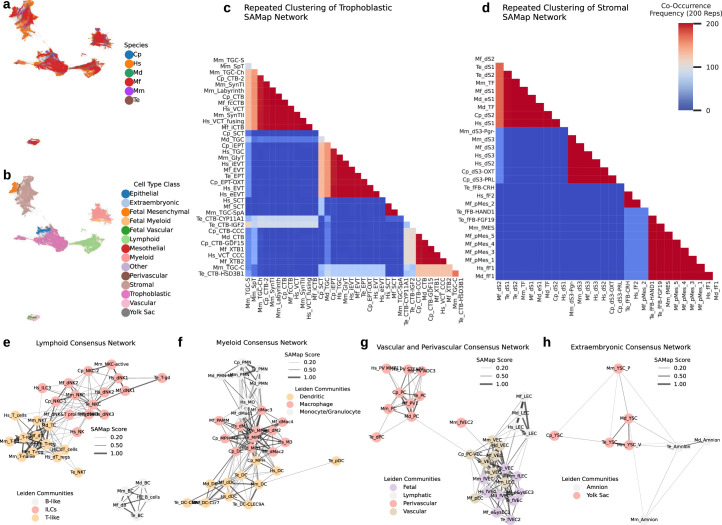
Cross-species cell type mapping. **a**. SAMap cross-species unified embedding colored by species of origin. **b**. SAMap embedding colored by cell type class. **c**. Co-occurrence matrix of trophoblast cell types in the same Leiden cluster across subsampled SAMap network iterations used to construct the network in Fig. 2c. **d**. Co-occurrence matrix of stromal cell types in the same Leiden cluster across subsampled SAMap network iterations used to construct the network in Fig. 3f. **e**-**h**. SAMap networks colored by Leiden community. Nodes are cell clusters, and edge weights are equal to mean mapping scores across 200 bootstrap runs. Edges with weights less than 0.15 are pruned for clarity in graphs **e**-**g**.

To assess cross-species cell type homology, we applied self-assembling manifold mapping (SAMap)^13^, which embeds cells from all species in a unified manifold (Fig. 1d and Extended Data Fig. 5a,b). Mapping scores between cell clusters showed consistent linkages between conserved cell types including smooth muscle, pericytes, leucocytes, endothelial and mesothelial cells (Supplementary Table 2). By contrast, trophoblast and decidual cell types—the functionally specialized and phylogenetically younger components of the fetal–maternal interface—displayed lower transcriptomic conservation, suggesting rapid evolution. This pattern motivated more targeted analyses of these cell types’ evolutionary history.

### Trophoblasts are transcriptomically and functionally diverse

Trophoblast cells are traditionally classified by location (for example, extravillous), phenotype (for example, glycogen trophoblast, syncytiotrophoblast) or ploidy (for example, giant cells), but whether these divisions reflect conserved cell type identities has remained unknown.

**Fig. 2:**
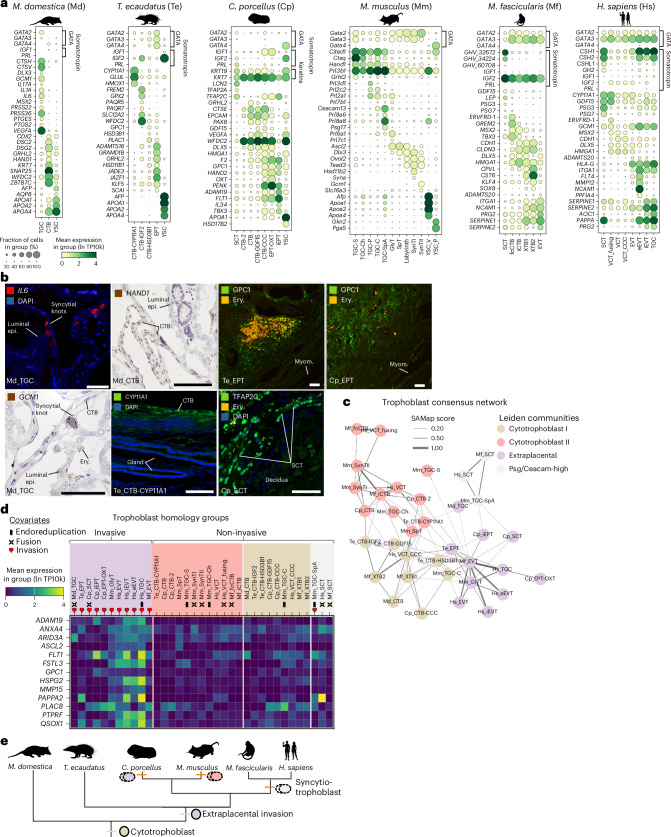
Trophoblast cell types and homology inference. **a**, Expression of trophoblast marker genes. **b**, Histological localization of select cell type markers from **a**. Epi., epithelium; myom., myometrium; ery., erythrocytes. Scale bars, 100 μm. **c**, Network of SAMap linkages between trophoblast cells, with edge thicknesses proportional to mapping scores. Edges with scores less than 0.15 are not shown for clarity. Nodes are coloured by Leiden community. **d**, Expression of select ‘pan-invasive’ genes in trophoblast of all species, grouped by SAMap homology group. Phenotypic covariates (endoreduplication, invasion, fusion) are annotated below cell type labels. Invasiveness groups by community, whereas fusion and endoreduplication are widespread. **e**, Summary cladogram of reconstructed events in trophoblast cell type evolution. CCC, cell column; CTB, cytotrophoblast; EPT/EVT, extraplacental/extravillous trophoblast; SCT, syncytiotrophoblast; TGC, giant cell; YSC, yolk sac cell. Silhouettes from PhyloPic under a Creative Commons licence: human, NASA (PDM 1.0); tenrec, Yan Wong (CC0 1.0); guinea pig, D.S. (CC0 1.0); opossum, D.S. (CC0 1.0); macaque, Jane Whitehouse (CC0 1.0); mouse, Michael Keesey (PDM 1.0).

**Extended Data Fig. 6:**
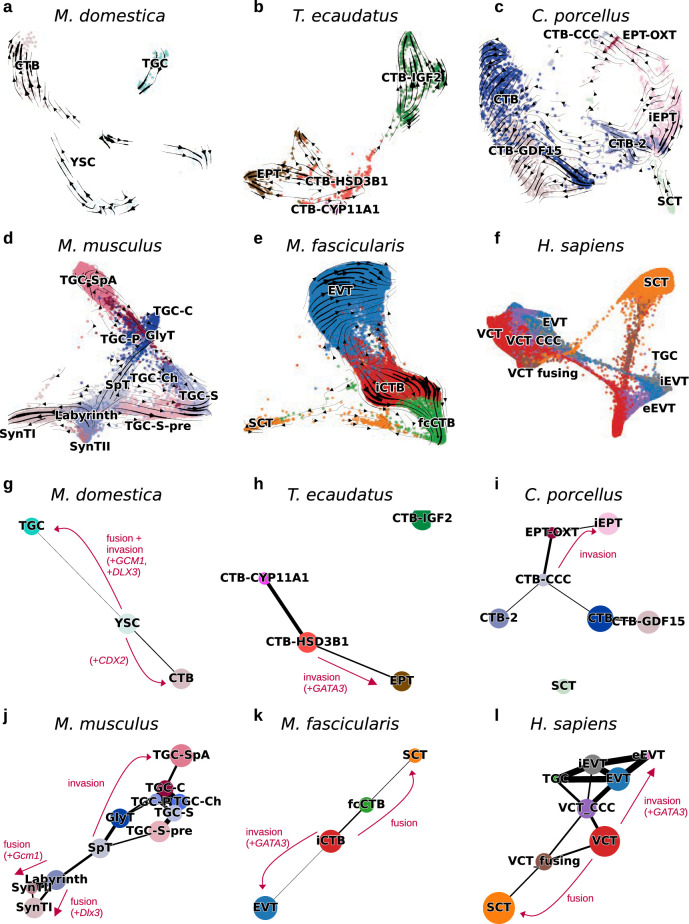
Inferred developmental trajectories of trophoblast cell populations. **a**-**f**. Force atlas embeddings of trophoblast cell types overlaid with RNA velocity (scVelo^45^) vector fields showing developmental directionality. **g**-**l**. PAGA connectivity graphs, where edge weight corresponds with transition probability. Thresholds for plotting edges were selected based on the density of the graphs: low (0.02; opossum and macaque) medium (0.10, guinea pig and tenrec) or high (0.20, human and mouse). Human data (**f**,**l**) lack velocity information but a spatially-verified developmental trajectory is available in the original publication^5^.

Single-cell atlases^5,8^ have identified a robust division of human and macaque trophoblast cells into three major classes: cytotrophoblast (CTB/VCT), syncytiotrophoblast (SCT) and extravillous trophoblast (EVT)^8^ (Extended Data Fig. 6). EVTs migrate interstitially (Hs_iEVT) into the decidua or endovascularly (Hs_eEVT) to replace spiral artery endothelium. Human and macaque share marker expression patterns: syncytiotrophoblast expresses placental-specific somatotropins (*CSH1-2* and *GH2* also known as *GHV*), *GDF15* and the syncytin *ERVFRD-1* during early fusion (Fig. 2a); cytotrophoblast expresses *CDH1* and *DLX5*; and extravillous trophoblast expresses *ITGA1*, *SERPINE1* and *SERPINE2* (Fig. 2a).

In the opossum, we identified two trophoblast populations (Fig. 2a). Cytotrophoblast (Md_CTB) expresses *WFDC2*, desmosome components (*DSC2* and *DSG2*), and transcription factors *ZBTB7C* and *HAND1*, the latter localized to mononuclear cells of the trophectoderm (Fig. 2b). These cells also express the t-SNARE *SNAP25* involved in vesicle fusion^14^, possibly with maternal apocrine secretory bodies (Extended Data Fig. 4a). Syncytial trophoblast includes giant cells of the trophectoderm (Md_TGC) termed ‘syncytial knots’; unlike murine giant trophoblast, these cells arise by fusion rather than endoreduplication^15^ (Fig. 2b). Syncytial knots express transcription factors *GCM1*, *MSX2* and *DLX3*, proteases (*CTSV*, *CTSH*, *PRSS22* and *PRSS36*), *VEGFA* and inflammatory mediators (*IL1A*, *IL6*, *IL17A*, *PTGES* and *PTGS2*). Yolk sac cells expressing apolipoproteins *APOA1*, *APOA2* and *APOA4*, and alpha fetoprotein *AFP*, were captured in all included species except primates^16^ (Fig. 2a), but only contribute to the primary placenta in opossum.

In the tenrec we identified one invasive and three non-invasive trophoblast populations (Fig. 2a). A steroidogenic cytotrophoblast population (Te_CTB-HSD3B1) expresses the progesterone synthase *HSD3B1* and the cortisol/cortisone converting enzyme *HSD11B1*, and is a likely progenitor of other trophoblast types (Extended Data Fig. 6). Another steroidogenic cytotrophoblast cluster (Te_CTB-CYP11A1) expresses *CYP11A1*, which we localized to the superficial trophoblast layer extending around the endometrium (Fig. 2b), and haem oxidase *HMOX1*, consistent with maternal haemoglobin breakdown reported in the trophoblast of other tenrec species^17^. A third cytotrophoblast cluster (Te_CTB-IGF2) expresses *WFDC2* and *IGF2*, progesterone receptors *PAQR5* and *PAQR7*, but lacks steroid biosynthesis enzymes. No fetal cell type expresses placental growth hormone or prolactin homologues; the only placental somatotropins are *IGF1* and *IGF2*. Trophoblast marked by *ADAMTS18*, *GATA3*, *PAPPA2* and *QSOX1* and cell invasion regulators *SCAI*^18^ and *ZEB1*^19^ (Fig. 2a,d) show striking similarity to extravillous trophoblast of the human and macaque; as the tenrec placenta lacks villi, we term them extraplacental trophoblast (Te_EPT). Cells immunoreactive for the proteoglycan GPC1, a marker of invasive trophoblast^20^ and angiogenesis^21^, surround the deepest maternal vasculature (Fig. 2b), suggesting interstitial invasion.

In the guinea pig, invasive extraplacental trophoblast (Cp_iEPT) expresses *ADAM19*, *FLT1*, *IL34* and *TBX3* (Fig. 2a), and a cluster enriched in *OXT* (Cp_EPT-OXT) is inferred to be developmental precursors to iEPT (Extended Data Fig. 6). Invasion is evident as trophoblast cells (KRT7^+^) are present in the decidua and surrounding maternal vasculature (Extended Data Fig. 4c), and GPC1^+^ invasive cells reach the myometrium (Fig. 2b). Cells from the subplacenta, a lineage-specific cytotrophoblast structure anchoring the placenta to the maternal decidua, included cells (Cp_CTB-CCC) sharing expression of proliferation regulators *DLX5*^22^ and *HMGA1* with human cell column cytotrophoblast (Hs_VCT-CCC), probably representing a progenitor population homologous to the human cell column^23,24^. Non-invasive cytotrophoblasts (Cp_CTB, Cp_CTB-2 and Cp_CTB-GDF15) express *CTSE*, *EPCAM* and *PAX8* (Fig. 2a). Syncytiotrophoblast (Cp_SCT) express three paralogues of prolactin, transcription factors *TFAP2A* and *TFAP2C*, and *LCN2*, a gene linked to invasion in human EVT^25^. Syncytiotrophoblast ‘tongues’, identified by morphology and TFAP2C expression (Fig. 2b and Extended Data Fig. 4d), invade into the decidua^24^. Giant cells were histologically evident (Extended Data Fig. 4e), but not captured transcriptomically.

Mouse trophoblast cells grouped into spongiotrophoblast, two types of syncytiotrophoblast, labyrinth cytotrophoblast, glycogen trophoblast and giant cells (Fig. 2a). Invasive trophoblasts are united by the expression of *Cdx2*, *Pcdh12*^26^, *Plac1* and *Tfap2c*. These include interstitially invasive *Prl6a1*^*+*^ *Prl7b1*^*+*^ *Prl7c1*^*+*^ glycogen trophoblast (Mm_GlyT)^27^, and endovascularly invasive spiral-artery-remodelling giant cells (Mm_TGC-SpA) expressing *Prl8a6*, *Prl8a8*, and a wide array of *Psg* and *Ceacam* genes. Giant cells were united by expression of *Cited1*^28^ and divided into *Prl2c2*^*+*^ *Prl3b1*^*+*^ *Prl3d1*^*+*^ *Ctsq*^*−*^ parietal (TGC-P), *Prl2c2*^*+*^ *Prl3b1*^*+*^ *Prl3d1*^*−*^ *Ctsq*^*−*^ canalicular (TGC-C)^29^ and *Prl2c2*^*−*^ *Prl3b1*^*+*^ *Prl3d1*^*−*^ *Ctsq*^*+*^ sinusoidal (TGC-S) and channel (TGC-Ch) populations (Fig. 2a). Two layers of syncytiotrophoblast lie in close proximity to maternal blood in the labyrinth, Mm_SynTI marked by *Hsd11b2* and *Syna*^30^, and Mm_SynTII marked by *Gcm1* and *Slc16a3*^31^.

### A eutherian radiation of invasive trophoblasts

To explore trophoblast relationships across species, we built a SAMap network and identified reciprocally linked cell clusters (Fig. 2c). The four resulting communities, representing putative homology groups, divided into one consisting of invasive cell types, two non-invasive groups, and one characterized by expanded *CEACAM* and *PSG* gene family expression (Fig. 2d).

The invasive trophoblast community included human and macaque EVT (Hs_EVT, Hs_eEVT/iEVT; Mf_EVT), tenrec extraplacental trophoblast (Te_EPT), and invasive rodent cells (Mm_GlyT, Cp_iEPT, Cp_SCT). These linkages were driven by proteases and known invasion-associated genes, including *ADAM19*, *MMP15*^32^, *PAPPA2*^33^, *ANXA4*^34^, *GPC1*^21^ and *PLAC8*^35^ (Fig. 2d and Supplementary Table 3), suggesting a conserved invasive program. Opossum syncytial knot cells (Md_TGC) arise as the most likely marsupial homologue to the invasive trophoblast of eutherian placentas. Guinea pig SCT is the only syncytial cell type in our study that invades and is mapping to invasive trophoblasts of other species.

Two other communities included non-invasive cytotrophoblast populations (Fig. 2d). The first linked putative developmental precursors across species—human villous cell column cells (Hs_VCT-CCC), guinea pig subplacental progenitors (Cp_CTB-CCC), opossum cytotrophoblast (Md_CTB) and tenrec steroidogenic cytotrophoblast (Te_CTB-HSD3B1)—consistent with PAGA connectivity analysis (Extended Data Fig. 6). The second consisted of diverse eutherian cytotrophoblast and labyrinth cell types and excluded the opossum. This pattern suggests that eutherian non-invasive trophoblast may derive from a single ancestral cytotrophoblast cell type retained in the opossum.

Phenotypes such as fusion and endoreduplication did not sort consistently into SAMap communities (Fig. 2d). For example, human trophoblast giant cells (Hs_TGC), derived from interstitial EVT^5^, mapped to extraplacental cells of other species rather than to mouse giant cells. Likewise, syncytiotrophoblasts lacked consistent mapping across species. Strong SAMap linkage occurred only between macaque and human SCT (Hs_SCT and Mf_SCT) and their fusion-stage progenitors (*ERVFRD-1*^*+*^ Hs_VCT_fusing and Mf_fcCTB; Fig. 2c), despite shared expression of syncytial transcription factors *GCM1* and *MSX2* between mouse SynTII and opossum TGC. Instead, primate syncytiotrophoblast grouped with mouse spiral-artery-remodelling trophoblast, driven by high *PSG* and *CEACAM* gene expression (Supplementary Table 3). As these gene families expanded independently in rodents and primates^36^, this probably reflects convergent evolution of glycogen-rich trophoblast rather than shared ancestry.

Overall, these patterns suggest that eutherian invasive trophoblasts descend from a cell type in the therian common ancestor with latent invasive potential (Fig. 2e), retained in *Monodelphis*. In Boreoeutheria, this invasive cell type radiated into interstitially migratory and artery-remodelling subtypes with diverse morphologies, including endoreduplication (Fig. 2d). We infer that beyond the recently diverged macaque and human, syncytiotrophoblast and giant trophoblast cell types arose independently in major mammalian lineages.

### Decidual cell diversity and a predecidual cell in *Tenrec*

Decidualization is the pregnancy-specific transformation of endometrium, including stromal fibroblast differentiation, arterial remodelling and lymphocyte recruitment. Decidualization is histologically observed only in mammals with haemochorial placentation^1^, and regulatory interactions among core decidual cell transcription factors are conserved among eutherians but absent in marsupials^37,38^, supporting its origin as a eutherian novelty. We used our cross-species atlas to assess whether the decidual stromal cell is a conserved cell type across eutherians and the extent of decidual diversity beyond the single prolactin-producing cell type canonically recognized in the field^39^.

**Fig. 3:**
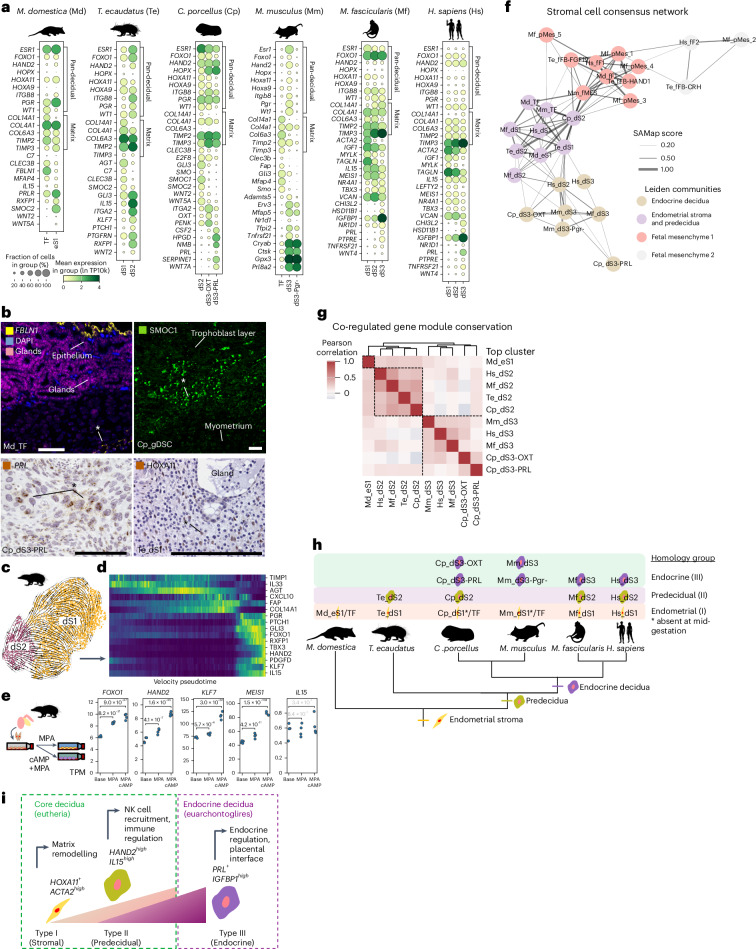
Cross-species comparison of decidual stroma. **a**, Marker gene expression of endometrial stromal and decidual cell types. **b**, In situ hybridization (italics) and immunohistochemical (upright) localization of select markers genes from **a**. Cells of interest are marked by an asterisk. Scale bars, 200 μm. **c**, Force atlas embedding of tenrec endometrial stromal cells with RNA velocity vectors overlaid. **d**, Expression of select velocity-associated genes in the dS1 → dS2 transition in all cells from **c**, ordered by increasing velocity-informed pseudotime. **e**, Expression of predecidual markers in primary tenrec uterine stromal cells cultured in vitro with deciduogenic stimuli. *P* values reflect PyDESeq2 Wald test *P* values after Benjamini–Hochberg correction for multiple testing. **f**, Network of SAMap mapping scores between stromal cell types, with edge weight proportional to mapping scores, coloured by community as identified by Leiden clustering of the graph. Edges with scores less than 0.15 are not shown for clarity. **g**, Correlation heatmap of cNMF gene expression programs expressed in decidual stromal cells, with a hierarchically clustered dendrogram on top showing division into two families. The opossum eS1 cell program (non-deciduate endometrial stromal cells) is included as an outgroup. **h**, Tree showing inferred evolutionary relationships of decidual cell types. **i**, Schematic of developmental relationships between decidual cell types and corresponding gene-regulatory signatures. Silhouettes from PhyloPic under a Creative Commons licence: human, NASA (PDM 1.0); tenrec, Yan Wong (CC0 1.0); macaque, Jane Whitehouse (CC0 1.0); mouse, Michael Keesey (PDM 1.0). Source data

Single-cell studies^4,5^ have revealed that the human and macaque decidual stromal cells fall into three broad categories. Type I stromal cells (dS1) are enriched in contractility- and myofibroblast-associated genes *ACTA2* and *TAGLN* (Fig. 3a). Predecidual cells^40^ (type II or dS2) express the NK-cell recruiting cytokine *IL15* and transcription factors *HAND2*, *MEIS1*, *TBX3* and *FOXO1* (Fig. 3a). Endocrine decidual cells (type III or dS3) uniquely express *PRL*^39^ and are enriched in *IGFBP1*, *PTPRE*, *CHI3L2*, *NR1D1* and *TNFRSF21* (Fig. 3a)*. IL15*^*+*^ *PRL*^*−*^ predecidual cells develop spontaneously during the secretory phase of the human menstrual cycle, and during the first wave of in vitro decidualization, whereas development of endocrine dS3 appears to be pregnancy-specific^41,42^. The conservation of this tripartite division between human and macaque prompted us to investigate possible homologues more broadly.

In the opossum, two stromal populations were identified. Peri-glandular^43^ endometrial fibroblasts (Md_eS1) express *SMOC2*, *WNT5A* and the relaxin receptor *RXFP1*. Myometrial fibroblasts (Md_TF) are enriched for *FBLN1*, *CLEC3B* and *MFAP4* (Fig. 3b). A subset of Md_eS1 expresses *HAND2* and *IL15*, markers of human predecidual cells thought to be eutherian-specific^44^, but these cells did not form a distinct cluster. This suggests that expression of *HAND2* and *IL15* is a developmentally accessible gene-regulatory state even in opossum stromal cells, but became stabilized into a robust cell type identity only in eutherians.

In the tenrec, two stromal populations were identified, which RNA velocity^45,46^ analysis inferred to constitute a developmental trajectory from *SMOC2*^*+*^ *CLEC3B*^*+*^ *COL14A1*^*+*^ endometrial fibroblasts (Te_dS1) to *IL15*^*+*^ *HAND2*^*+*^ predecidual-like cells marked by hormone receptors *ERBB4*, *RXFP1*, *ESR1* and *PGR*, and decidual transcription factor *KLF7* (Te_dS2) (Fig. 3c,d). Localization of HOXA11, a shared marker of both fibroblasts, to peri-glandular endometrial stroma (Fig. 3b) supports their homology with the eS1 of opossum. IL-15 is a chemokine for uterine natural killer (NK) cell recruitment^4^, and indeed NK cells are abundant in the tenrec (Fig. 1c). Treatment of stromal cells isolated from the pregnant uterus of *T. ecaudatus* with deciduogenic stimuli 8-Br-cAMP and medroxyprogesterone acetate (MPA) for 6 days elicited upregulation of these predecidual markers (Fig. 3e). As this treatment is sufficient to generate endocrine decidua in human cells^39^, we concluded that the predecidual cells observed represent this species’ full decidual response. Together, these findings suggest that tenrec uterine stroma persists in a predecidual state homologous to the cells of the human luteal-phase endometrium rather than progressing to endocrine decidual cells.

The guinea pig decidual stroma contained one extracellular matrix-remodelling and two endocrinologically active populations. All three express *HOXA11*, *HAND2*, *ESR1*, *PGR* and the rodent-specific decidual marker *HOPX*^47^ (Fig. 3a). Endocrine decidual cells divide into *OXT*^*+*^ (Cp_dS3-OXT) and *PRL*^*+*^ (Cp_dS3-PRL) clusters, with the latter located surrounding maternal spiral arteries (Fig. 3b). The most abundant population of stromal cells (Cp_dS2) was *SMOC2*^*+*^ *WNT5A*^*+*^ and showed SAMap affinity to primate predecidual cells. Based on location near necrotic patches (Fig. 3b and Extended Data Fig. 4h) and expression of the cell cycle gene *E2F8* implicated in endoreduplication^48^, this latter population is possibly the source of giant decidual cells described histologically in the guinea pig^24^.

The mature mouse decidua contained a single endocrine cell type (Mm_dS3) marked by *Prl8a2*, *Pgr*, *Esr1*, and *Hand2* and *Col14a1*^*+*^ myometrial fibroblasts (Mm_TF). A *Pgr*^*−*^ *Esr1*^*−*^ population of decidual cells (Mm_dS3-Pgr^−^) showed reduced expression of *Hand2* and *Hoxa11* but maintained expression of *Prl8a2*, stress-related genes *Cryab*, *Gpx3* and *Ctsk*. These cells match what a previous study identified as postmature decidua^49^ and share similarities with progesterone-resistant human decidual cells^50^. Mouse dS3 expressed six genes recently discovered to have maternally biased parent-of-origin imprinting^51^—*Adamts5*, *Erv3*, *Mfap5*, *Tfpi2*, *Tnfrsf11b* and *Tnfrsf23* (Fig. 3a): these genes are tumour suppressors^52,53^ and may represent mechanisms to regulate trophoblast invasion.

### Stepwise evolution of the uterine decidua

SAMap network analysis of stromal cells resolved two communities of maternal stromal cells and two of fetal mesenchymal cells (Fig. 3f). Among maternal cells, one community grouped endocrine (type III) decidual cells, including the two endocrine decidual cell populations of the guinea pig (*OXT*^*+*^ and *PRL*^*+*^), mouse *Pgr*^*+*^ and *Pgr*^*−*^ decidual cells, and human and macaque *PRL*^*+*^ cells. The other included non-decidualized fibroblasts (opossum eS1 and eutherian dS1), myometrial fibroblasts from opossum and mouse, guinea pig dS2, and macaque and tenrec predecidual cells. Human predecidual cells (Hs_dS2) showed affinity to both predecidual and endocrine groups, but this distinction probably reflects developmental continuity rather than evolutionary divergence.

To further assess gene-regulatory conservation, we compared co-regulated gene modules within decidual cells across species. Hierarchical clustering of gene expression programs active in predecidual dS2 and endocrine dS3 cells showed robust separation into two families (Fig. 3g): predecidual modules consistently included *MEIS1*, *WT1*, *ESR1*, *PGR* and *EGFR*, and were active in human, macaque, tenrec and guinea pig. Endocrine gene-regulatory modules showed co-regulation between *PRL*, *LUM*, *DCN*, *PPIB* and *DUSP1*, and were active in the endocrine decidua of mouse, guinea pig, human and macaque. These genes also arose as top linking genes in SAMap analysis of dS2–dS2 and dS3–dS3 pairs, respectively (Supplementary Table 4). Predecidual and endocrine gene expression modules tended to be anticorrelated (Fig. 3g), suggesting that predecidual and endocrine decidual gene expression programs have robustly distinct regulatory underpinnings.

These results support a model in which decidual stromal cell evolution (Fig. 3h) and development (Fig. 3i) both involve two stages. Contractile endometrial fibroblasts (type I) are conserved across therian mammals. In eutherians, these gave rise to predecidual cells, which secrete immunomodulatory peptides, followed by the later emergence of endocrine decidual cells (type III), which secrete PRL and other growth factors. Type II decidual cells represent a novel eutherian stromal cell type derived from mesenchymal cells of the ancestral therian endometrium by upregulation of *HAND2* and *IL15*^54^. The presence of predecidual cells in the tenrec but not in the mouse suggests that type II cells arose early in eutherian evolution but were lost or modified in some lineages, or are restricted to early stages of mouse pregnancy not sampled here. In rodents, *Il15* is produced by macrophages, which may take over the immune-recruitment function of the predecidua. Together, our findings suggest that the decidual cell type first emerged in a predecidual *IL15*^*+*^ *PRL*^*−*^ state, followed by the addition of endocrine decidual cells later within crown Placentalia.

### Co-evolutionary dynamics of cell communication

To identify signalling co-evolution between fetal and maternal cell types, we inferred ligand–receptor interactions from single-cell transcriptomes and traced their evolutionary history across Theria.

#### Trophoblast and decidual cells show signalling integration

**Fig. 4:**
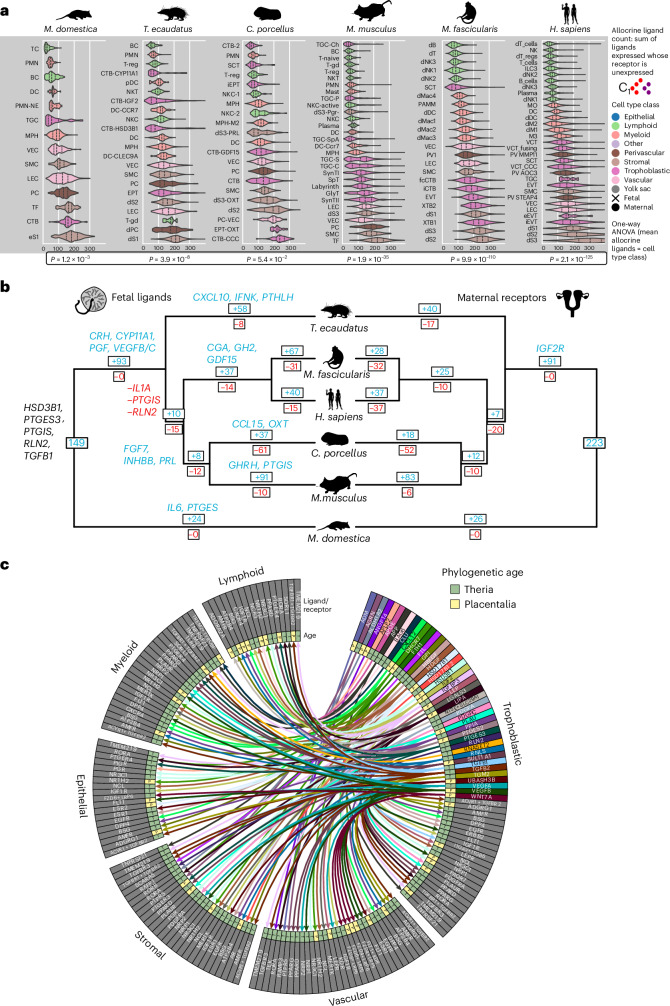
Analysis of ligand–receptor signalling and reconstruction of evolutionary changes. **a**, ALCs (ligand expressed, receptor unexpressed) for genes encoding secreted signals grouped by cell type and species, and coloured by cell type class. Vertical lines represent the first quartile, median and third quartile. *P* values of ANOVA relating ALCs to cell type class in each species are plotted. *F* statistics: *M. domestica*, 6.09; *T. ecaudatus*, 12.28; *C. porcellus*, 2.43; *M. musculus*, 44.77; *M. fascicularis*, 177.62; *H. sapiens*, 147.74. **b**, Co-evolution of binary expression of signals by placental cells (left tree) and cognate maternal receptors (right tree) inferred from maximum parsimony reconstruction, with select gains and losses plotted in blue and red, respectively. **c**, Reconstructed secreted signalling interactions in the Placentalia common ancestor, coloured by phylogenetic age of gene expression (P, eutherian novelty; T, retained from ancestral therian), partitioned by cell type class and subset to only those derived from the trophoblast. Silhouettes from PhyloPic under a Creative Commons licence: human, NASA (PDM 1.0); tenrec, Yan Wong (CC0 1.0); macaque, guinea pig, D.S. (CC0 1.0); opossum, D.S. (CC0 1.0); Jane Whitehouse (CC0 1.0); mouse, Michael Keesey (PDM 1.0). Source data

**Extended Data Fig. 7:**
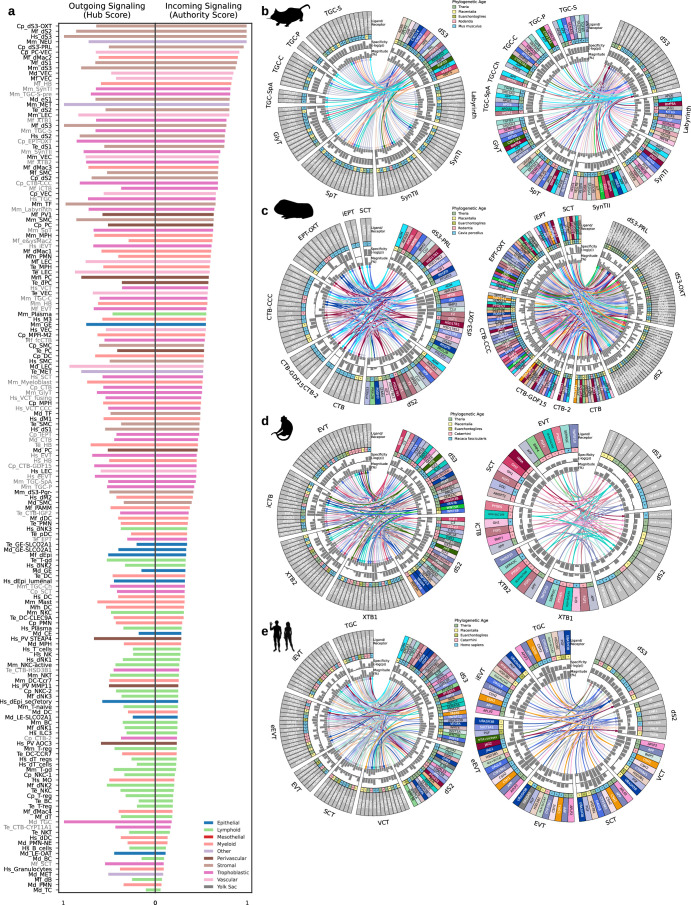
Extended cell-cell communication analysis. **a**. Graph statistics of all cell types within their respective species’ cell-cell signaling networks. Kleinberg hub scores (a measure of outgoing signaling) are shown on the left and Kleinberg authority scores (a measure of incoming signaling) on the right. Graphs are weighted based on the product of expression values of ligand and receptor. **b**-**e**. Evolutionarily novel signaling interactions between trophoblast and decidual cell types in Euarchontoglires. Left plots show decidual→trophoblast signals and right plots show trophoblast→decidual signals. Ligand and receptor complexes are annotated by the phylogenetic age of their expression as inferred by maximum parsimony ancestral state reconstruction. Outer bar graphs represent LIANA+ specificity rank p-values and inner magnitude bar graphs represent the proportion of cells in the cluster expressing the ligand or receptor. b: M. musculus, c: C. porcellus, d: M. fascicularis, e: H. sapiens. Silhouettes from PhyloPic under a Creative Commons licence: human, NASA (PDM 1.0); tenrec, Yan Wong (CC0 1.0); macaque, Jane Whitehouse (CC0 1.0); mouse, Michael Keesey (PDM 1.0).

The evolution of placentation requires functional integration between fetal and maternal cells^55,56^. To approximate functional integration, we calculated the number of non-autocrine signalling interactions each cell engages in, which we term the allocrine ligand count (ALC) (Methods). ALCs varied across major cell type families (analysis of variance (ANOVA) *P* < 0.05 in all species except *C. porcellus*) (Fig. 4a). Across species, decidual stromal cells and invasive trophoblasts (EVT, EPT, Mm_GlyT, Md_TGC) displayed the greatest degrees of allocrine signalling, whereas syncytiotrophoblast and peripheral immune effector cells (BC, TC, NKC, PMN) showed the fewest^56^ (Fig. 4a). Network analysis^57^ supported these patterns (Extended Data Fig. 7): *Monodelphis* TGC and human endocrine decidual cells act as hubs in their respective signalling networks, whereas lymphoid cells from all species remain poorly integrated. These results suggest that fetal and maternal cell types that make direct contact evolved to function as central nodes in uteroplacental signalling networks.

#### Ancestral state reconstruction of cell–cell signalling networks

We reconstructed ancestral gene expression states at key nodes in the mammalian phylogeny to infer signalling network evolution. Using parsimony-based ancestral reconstruction (Methods), we identified 149 signals that the placenta of the first viviparous mammal probably sent to its mother, including relaxin *RLN2*, platelet-derived growth factors *PDGFA* and *PDGFC*, prostaglandin E2 via *PTGES3*, and *TGFB1* and *TGFB2* (Fig. 4b). The Placentalia lineage saw gains of 93 additional ligands, including placental growth factor *PGF*, vascular growth factors *VEGFB* and *VEGFC*, and the insulin-like growth factor *IGF2*. Within Euarchontoglires, several cytokines and chemokines, including *CCL2* and *CCL4*, evolved as fetal signals, whereas others, including *IL1A*, *TNF* and neutrophil chemoattractant *CXCL2*, were lost, suggesting a reduction of placental inflammatory signalling after the divergence of eutherians from marsupials. The two rodents in our sample shared derived placental ligands including *FGF7*, *IL34* and *PRL*, whereas primates shared novel growth hormone paralogues.

To identify cellular targets of these signals, we re-performed ancestral state reconstruction at the resolution of cell type classes. The cell-class-resolved reconstructed fetal–maternal signalling network of the placental mammal common ancestor (Fig. 4c) reveals that the early eutherian trophoblast had a potential to activate receptors in maternal stromal cells via ligands known to promote decidual development, like prostaglandin and relaxin^39^, and to communicate with maternal vascular, epithelial and immune cells via steroid hormones and *IGF2*. As novel decidual and placental cell types evolved, so did signalling pathways: the gain of decidual *PRL* expression in rodent and primate endocrine decidual cells enabled signalling to trophoblast cells that ancestrally express *PRLR*, and in Catarrhini established an autocrine loop by the gain of decidual *PRLR* expression (Extended Data Fig. 7). In rodents, a gain of trophoblast *KITLG* expression and decidual *KIT* expression enabled signalling via stem cell factor, and in both human and mouse, new channels of *WNT* signalling emerged between decidual and trophoblast cells by coordinated ligand and receptor expression gains (Extended Data Fig. 7). In sum, the evolution of diverse novel fetal trophoblast cell types as well as the diversification of maternal endocrine decidual cells in Euarchontoglires enabled novel channels of ligand–receptor communication.

#### Fetal and maternal signalling show disambiguation

**Fig. 5:**
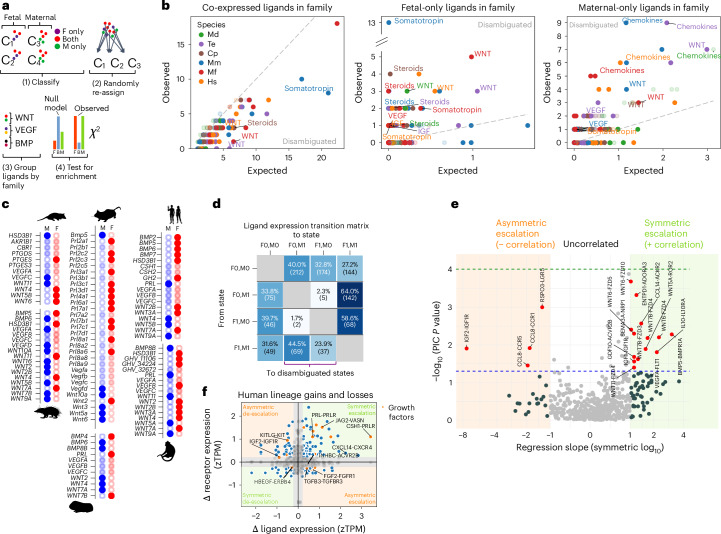
Tests of two evolutionary hypotheses for evolutionary dynamics of fetal–maternal communication, disambiguation^9^ and escalation^10^. **a**, Design for disambiguation test. F, fetal; M, maternal. **b**, Observed versus expected numbers of co-expressed, fetal-only and maternal-only ligands, by ligand family. Points with solid colour have both *P* < 0.05 and Benjamini–Hochberg false discovery rate *q* < 0.05. **c**, Disambiguation status of select ligands. Blue, maternal-only; red, fetal-only; faint, co-expressed. **d**, Transition matrix of expression of secreted signalling ligands in fetal and maternal cell types of the six-species phylogeny, coded as two binary states for fetal and maternal expression, respectively. Percentages are normalized by row. **e**, Volcano plot showing co-evolutionary coupling of ligand and receptor expression magnitude. Two-sided *P* values testing whether the slope of phylogenetically independent contrasts between ligand and receptor differs significantly from zero are plotted on the vertical axis, and ordinary least squares regression slopes of zTPM expression values of ligand and receptor are plotted on the horizontal axis. Pairs with strong evolutionary coupling fall into the shaded orange (negative correlation) and green (positive correlation) regions, and those with stronger statistical support are plotted in red. The blue dashed line marks a *P* value cutoff of 0.05, whereas the green dashed line marks a Bonferroni-adjusted *P* value cutoff of 0.05/*n*_pairs tested_. **f**, Inferred evolutionary changes to ligand and corresponding receptor expression in humans with respect to the human–macaque common ancestor (Catarrhini). Silhouettes from PhyloPic under a Creative Commons licence: human, NASA (PDM 1.0); tenrec, Yan Wong (CC0 1.0); macaque, Jane Whitehouse (CC0 1.0); mouse, Michael Keesey (PDM 1.0). Source data

Signalling theory predicts that fetal–maternal signalling systems evolve towards maternal-only or fetal-only expression to reduce fetal manipulation of maternal physiology^9^. Selection against co-expression of ligands would manifest as a disambiguation pattern, with restriction of gene expression to fetal or maternal cells exclusively. To quantify disambiguation, we compared the observed number of fetal-only and maternal-only expressed ligands from different signalling families (for example, WNT and, NOTCH) against a null model based on random assignment of ligands to cell types regardless of origin (Fig. 5a,b and Methods). Consistent with predictions, co-expression of ligands was lower than expected by our null model (Fig. 5b). Disambiguation was most pronounced in WNT, steroid, FGF and various immunomodulatory ligand families (Fig. 5b).

Gene duplication contributes to disambiguation by the origination of placenta- or decidua-specific paralogues. In the murine lineage, the prolactin gene family includes 26 members and a strongly disambiguated expression pattern with 10 fetal-specific paralogues (Fig. 5c). In human and macaque, duplication of the *GH1* locus has produced placenta-specific paralogues, whereas *PRL* is maternal-only (Fig. 5c). Growth factors essential to tissue homeostasis, such as *VEGFA-D*, *PDGFA-B* and *CSF1*, showed overwhelmingly bilateral expression (Fig. 5c). For example, extreme production of the angiogenic factor *VEGFA* by the opossum trophoblast can be interpreted as a nutrient-soliciting signal. The disambiguation hypothesis^9^ predicts that in an evolutionary stable state, the mother should silence her own copy or utilize a different VEGF paralogue, yet all three VEGF paralogues were co-expressed. *VEGF* and *PDGF* gene families originated at least 370 Myr ago and have not duplicated after the divergence of tetrapods^58^. The absence of disambiguation therefore cannot be explained by lack of time for tissue-specific expression to evolve, but may instead be explained by functional constraints. Disambiguation could also be achieved by utilization of a splice variant of VEGFA with differential signalling properties, as documented with the *VEGF*_*111*_ isoform in another marsupial, the fat-tailed dunnart, as well as the rat and viviparous skinks^59^.

We used our reconstructed ancestral states to trace evolutionary events of novel disambiguated ligand production across the six-species tree (Fig. 5d). Out of a total of 1,023 character state changes, 492 resulted in disambiguated states. Fetal-only signalling resulted more frequently from gains of placental expression than from losses of maternal expression (174 versus 37 instances). The latter type of transition has been proposed to enable gestational drive^60^, and ligands following this pattern in the human lineage include *INHBA* and *BMP2*.

#### Testing the escalation hypothesis

**Extended Data Fig. 8:**
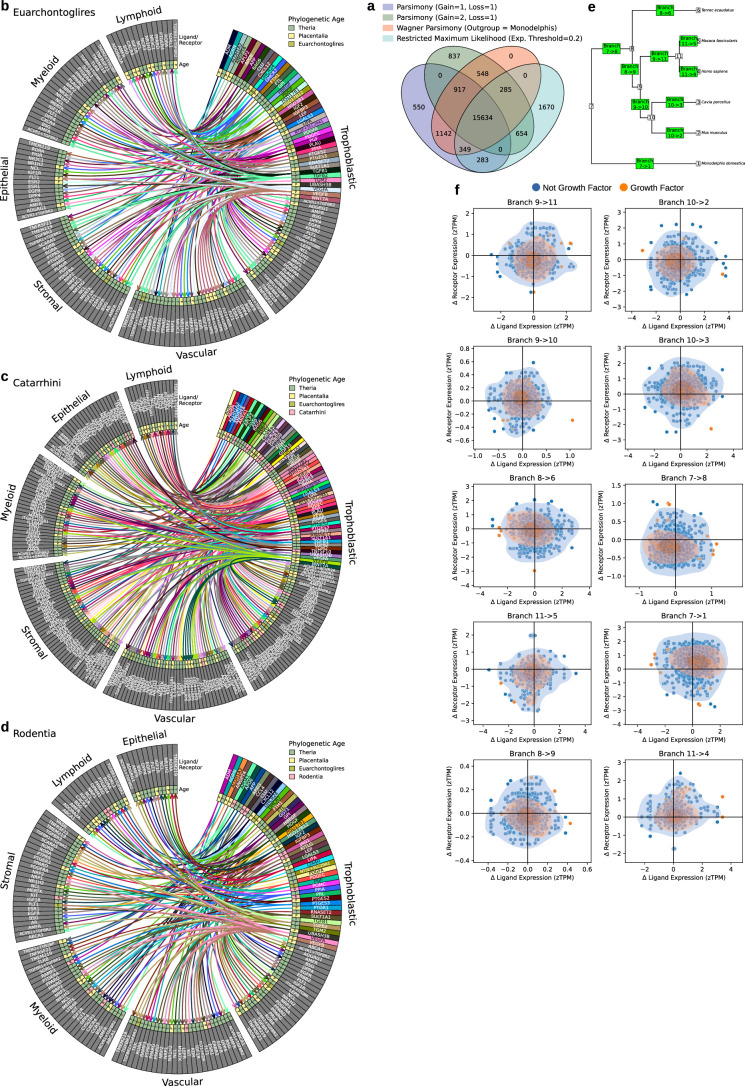
Ancestral state reconstruction of ligand-receptor signaling. **a**. Comparison of inferred ancestral state configurations between four ancestral state reconstruction methods (see Methods). **b**-**d**. Cell-cell signaling networks from trophoblast to maternal cells in common ancestral nodes Euarchontoglires (**b**) Catarrhini (**c**) and Rodentia (**d**). **e**. Phylogenetic tree of species sampled with numbering of internal and terminal nodes as well as internal branches. **f**. Inferred changes in inferred expression magnitude of placental ligands and cognate maternal receptors of secreted signals along internal branches of the phylogeny. Asymmetric escalation and de-escalation events fall into the lower-right and upper-left quadrants, respectively, while uncorrelated changes cluster around the center. Source data

Parent–offspring conflict theory predicts an asymmetric evolutionary arms race^61^ in which gains to fetal signalling strength are matched by reduced maternal responsiveness^10^. We tested this by modelling co-evolution of fetal ligand and maternal receptor expression across the phylogeny using phylogenetically independent contrasts^62^ (Fig. 5e). Reconstructed evolutionary changes along internal branches of the tree did not show a global trend towards escalation (Extended Data Fig. 8). Some pairs, most notably the well-studied gene pair *IGF2*–*IGF1R*^63^, showed negative correlation consistent with antagonism, whereas others such as *VEGFA*–*FLT1* and *IL10*–*IL10RA* showed positive correlation, possibly reflecting coordinated regulation of vascularization and immunomodulation in multiple eutherian lineages. After correction for multiple testing, the aforementioned gene pairs remained below or only marginal to statistical significance, which could be due to limited taxon sampling leading to low power to detect a correlation signal. In the branch leading from the human–macaque ancestor to humans, the growth factors *TGFB3* and *FGF2* showed asymmetric escalation relative to their receptors, whereas *KITLG–KIT* and *IGF2–IGF1R* showed de-escalation (Fig. 5f). The expansion of fetal growth hormone paralogues with strong PRLR activating affinity, *CSH1* and *CSH2*^64^, coincided with increased maternal *PRLR* expression.

Our findings reveal a history of fetal–maternal communication shaped by a mixture of conflict, cooperation and constraint. While some signalling pairs like *IGF2–IGF1R* exhibit antagonistic dynamics, asymmetry does not characterize fetal–maternal cell signalling as a whole. That said, our transcriptomic analysis does not exclude antagonistic dynamics at the post-transcriptional level, as in the case of decoy receptors^63^, altered binding affinities or chemical properties of signal peptides such as half-life^65^.

## Discussion

The eutherian fetal–maternal interface arose through three major evolutionary innovations—endometrial decidualization, the development of a distinct placental organ and the origin of trophoblast invasion^2^. Here, by comparing cell type composition and cell–cell signalling networks of species spanning these transitions, we reveal how trophoblast and decidual cell type diversity and signalling have evolved.

Cross-species mapping suggests that eutherians underwent a marked expansion in trophoblast diversity. We infer that the therian ancestor, like the extant opossum, had two trophoblast cell types, one homologous to eutherian cytotrophoblast and the other specialized in signalling to the mother with latent invasive potential. Invasive trophoblast populations across mammals share a conserved gene signature, suggesting that the invasive/non-invasive phenotypic divide reflects an ancient divide in trophoblast cell type identity rather than convergent evolution. Superficial placentation in marsupials is more likely due to brief fetal–maternal contact and lack of receptive decidua than absence of trophoblast functions for invasion.

**Extended Data Fig. 9:**
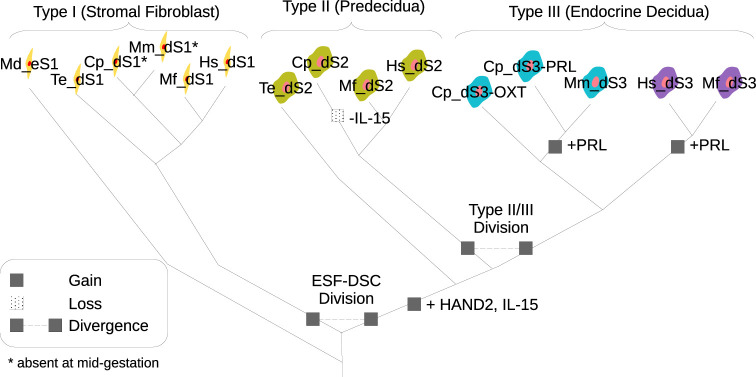
Cell type phylogeny showing evolutionary relationships between endometrial stromal cell types. Major phenotypic changes are marked as boxes. Modeled after (ref. ^66^).

Endometrial stromal cells partition into homology groups of different phylogenetic ages, indicating a stepwise acquisition of decidual cell diversity in eutherians (Fig. 3h,i and Extended Data Fig. 9). The discovery of an *IL15*^*+*^ predecidual cell in *Tenrec* with similarity to human type II decidual cells suggests that this cell type predates the 99 Myr *Tenrec–Homo* divergence. Endocrine (*PRL*^*+*^) decidual cells appear to have arisen only later in eutherian evolution, restricted in our sampling to humans and rodents (Euarchontoglires) and lacking in *Tenrec*. Future investigation of more mammals from Afrotheria and Xenarthra will allow the endocrine decidual cell’s origins to be more thoroughly untangled.

**Extended Data Fig. 10:**
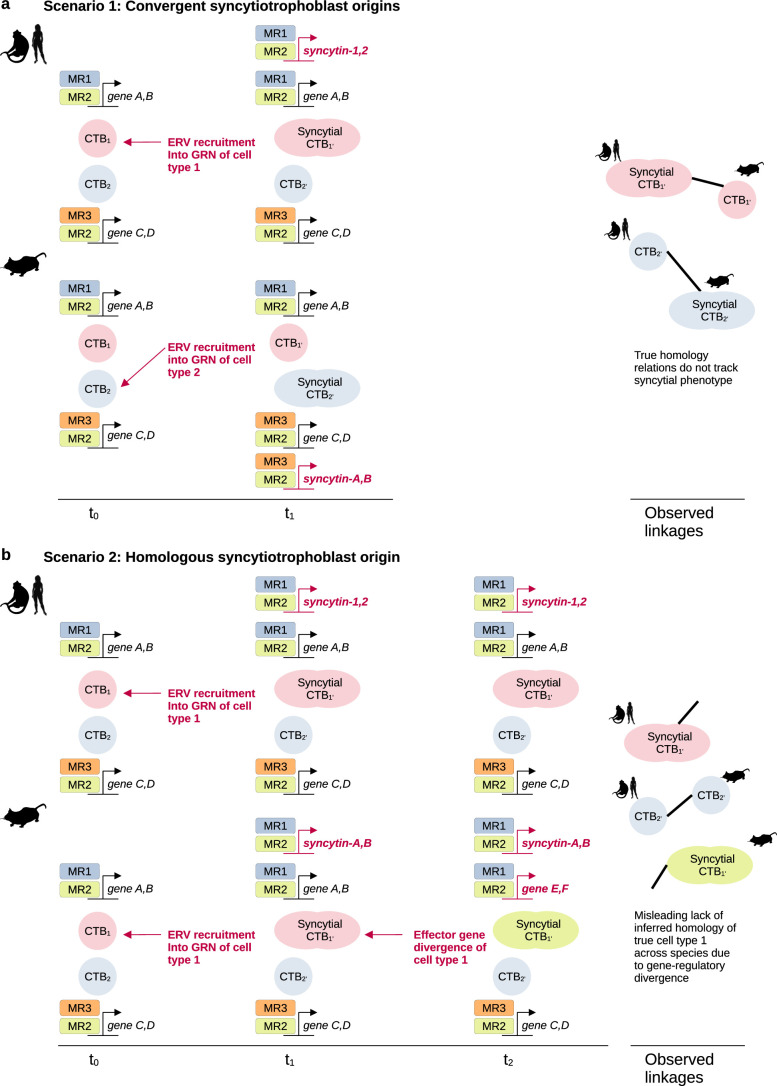
Comparison of two possible scenarios for the evolution of trophoblast syncytialization. **a**. In the convergent syncytiotrophoblast origins scenario, endogenous retroviral (ERV) envelope genes are into the genome of different mammalian lineages in locations which fall under the control of regulatory genes (MR) in different placental cell types’ gene-regulatory networks (GRN). This leads to non-similarity between syncytial cell types in cross-species transcriptomic comparison (for example SAMap) which should be interpreted as true non-homology. **b**. in the single syncytiotrophoblast origin scenario, ERV envelope genes were recruited into the GRNs of the same (homologous) progenitor cell type, which subsequently underwent transcriptomic divergence and/or functional specialization in different lineages (recruitment of genes E and F in the figure), leading to lack of similarity in extant species which should be interpreted as functional divergence rather than non-homology. Silhouettes from PhyloPic under a Creative Commons licence: human, NASA (PDM 1.0); tenrec, Yan Wong (CC0 1.0); macaque, Jane Whitehouse (CC0 1.0); mouse, Michael Keesey (PDM 1.0).

Our findings align with a model of cell type evolution that distinguishes between broad transcriptomic ‘cell type identities’ and functional programs or ‘apomeres’^66^, a pattern seen in neuron^67^, muscle^68^ and photoreceptor^69^ evolution. Decidual and trophoblast diversification has involved evolution of novel functional programs, like cell fusion and hormone production, frequently driven by insertion of retroviral and transposable elements^70^. On the fetal side, cell fusion arose through repeated insertions of retroviral envelope genes in opossum, tenrec, guinea pig, muroid and primate lineages^71–73^. Acquisition of this apomere by different ancestral trophoblast cell types could explain the transcriptomic dissimilarity we observe (Extended Data Fig. 10), as well as the rich diversity of secondary syncytiotrophoblast functions—inflammatory and growth factor signalling from the giant syncytial cells of the opossum, invasion in the guinea pig, vascular interfacing in the mouse, and gonadotropin production in the human. On the maternal side, prolactin expression by endocrine decidual cells is likewise associated with transposable element insertions upstream of the prolactin promoters in rodents and primates^74^. Identifying which of the cell populations we identify here correspond to developmentally robust cell type identities, and the potential for parallel cell type evolution, will require further targeted investigation.

Theoretical models have long been advanced for the co-evolutionary signalling dynamics between the fetus and mother^10^. Our test of the disambiguation hypothesis^9^ revealed consistent enrichment of fetal or maternal specificity in ligand production, driven by lineage-specific gene duplications, including prolactin paralogues in rodents and growth hormone paralogues in primates^75^. Future work will be required to identify genetic mechanisms of disambiguation, including the possible role of fetal-specific enhancers^70^. The escalation hypothesis predicts that increased fetal ligand production should be complemented by reduction in corresponding receptor expression by the mother. Only a select few ligand–receptor pairs in our sample followed this dynamic; the strongest example being *IGF2*, the best established^76^. This suggests that IGF2 may be an edge case rather than a universal rule. Finally, we used ancestral state reconstruction to trace the co-evolutionary history of ligand–receptor signalling, and found that evolutionary turnover of ligand and receptor pairs has continued throughout eutherian diversification, not solely with the initial origin of invasive placentation in the stem lineage.

Several limitations to our study should be acknowledged. Inference of ligand–receptor signalling from RNA expression may underestimate true signalling events due to sequencing drop-out or incomplete genome annotations, which could inflate the number of species-specific signalling innovations inferred. Differences in gestation length and placental physiology between our six species limit the comparability of gestational stages and as such our ability to deduce the developmental hierarchy of trophoblast within each species’ placenta was limited (Extended Data Fig. 6). Nevertheless, the outlines of a multistage history of cell type and cell phenotypic evolution can clearly be seen.

The fetal–maternal interface has become a leading model for single-cell genomics and ligand-mediated cell signalling^3–5^. The atlases presented here demonstrate that long-standing evolutionary questions about parent–offspring co-evolution can now be addressed at cellular and molecular resolution. Mechanisms behind novel signal evolution, cell type integration and fetal–maternal disambiguation will require future investigation. As high-throughput genomics of non-traditional model species becomes more technologically accessible, greater taxon sampling will allow the phylogenetic analyses we introduce here to expand in precision and scope.

The comparative framework we introduce here for cell–cell communication holds potential for broader application. Similar approaches could illuminate the co-evolution of cell types within other complex tissues and symbiotic systems, an avenue opened by recent advances in single-cell transcriptomics of multicellular parasites^77^ and commensals^78^.

## Methods

### Animal husbandry

Pregnancy samples were taken at gestation points after the establishment of the definite placenta and, in the case of guinea pigs, before the luteal–placental shift in progesterone production. Time points chosen were in opossum day 13.5 of 14.5 total gestation, in tenrec day 28–29 of 56, in guinea pig day 30.5 of 62 and in mouse days 11.5–15.5 of 19.5 days gestation. These were integrated with a human atlas generated from 4 to 13 weeks, roughly approximate days 28–91 out of a 280-day gestation^5^, and macaque samples generated from stages ranging from 20 to 140 of a 162-day gestation^8^.

*M. domestica* were raised in a breeding colony at Yale University according to established technical protocols^79^ and ethical protocols approved by the Yale University Institutional Animal Care and Use Committee (2020-11313). Male and female animals were housed separately after reaching 3 months of age, at which point female opossums were moved to the male room for sexual preconditioning by exposure to male pheromones. Breeding was attempted after 6 months. During breeding, non-cycling female opossums were introduced to the male room for 1 day, and then subsequently swapped into the used cage of a prospective male partner for 5 days. After this period, both individuals were placed into a breeding cage and video recorded to assess the time of copulation. If multiple copulations were observed, the first was always used to calibrate 0 dpc (days post copulation); samples were taken 1 day before parturition at 13.5 dpc (*n* = 2).

*T. ecaudatus* were maintained in a breeding colony at the University of Nevada, Las Vegas according to approved University of Nevada, Las Vegas Institutional Animal Care protocols. Individuals in the colony descend from a population of 40 wild-caught animals imported from Mauritius in June 2014. Animals were mated and mid-gestation was sampled between 28 and 29 days (*n* = 3) following the first exposure of females to males.

*C. porcellus* (Charles River) were maintained at the University of Vienna according to Institutional Animal Care protocols, on standard chow and water ad libitum. The oestrus cycle was monitored by examination of vaginal membrane opening and the animals were mated in oestrus at 3–4 months of age, and video recorded to detect copulation. Mid-gestation samples (*n* = 2) were collected at 30.5 dpc.

*M. musculus* (C57BL/6 J) were maintained at the University of Vienna on standard chow and water ad libitum according to University Institutional Animal Care protocols. Animals of age 2.5–4 months were used for the experiments. The oestrus cycle was monitored by vaginal swabbing, and females were mated when approaching oestrus. Copulation was confirmed by the presence of a copulatory plug. The start of gestation is counted from the midnight of the night preceding the detection of copulatory plug. Mid-gestation samples (*n* = 2) were collected 15.5 dpc and supplemented with published data from 11.5–14.5 dpc (*n* = 9) described below.

### Single-cell RNA sequencing

Uteri were dissected into phosphate-buffered saline (PBS), separated from the cervix and fallopian tube, and opened longitudinally. Embryos and directly attached extraembryonic membranes were removed via severing of the umbilical cords. In the guinea pig, the labyrinth was removed prior to sample preparation. Tissue samples were kept at 4 °C for approximately 30 min until they could be dissociated into a single-cell suspension. Owing to necessity, *Tenrec* samples were shipped in RPMI medium on ice overnight and dissociated the next day.

All tissue was processed as follows, with small species-specific modifications as noted. Tissue was minced with a scalpel into ~1-mm^3^ cubes in 2 ml of digestive solution containing 0.2 mg ml^−1^ Liberase TL (05401020001, Sigma). Tissue suspensions were heated at 37 °C for 15 min and then passed ten times through a 16-gauge needle attached to a 3-ml syringe. This incubation and needle passage process was repeated two more times, with the substitution of an 18-gauge needle. Finally, 2 ml of charcoal-stripped fetal bovine serum (100-199, Gemini) was added and the suspension was immediately passed through a 70-μm cell strainer then a 40-μm cell strainer. The flowthrough was pelleted by centrifugation at 500*g* for 5 min and cells were resuspended in 1× ACK red blood cell lysis buffer (A1049201, Thermo Fisher), incubated at room temperature for 5 min, centrifuged again at 500*g* and resuspended in phosphate-buffered saline containing 0.04% bovine serum albumin (A9647, Sigma). At this point, cells were examined on a haemocytometer to gauge concentration and check for debris and cell death using trypan blue stain (15250061, Thermo Fisher).

Mouse tissue was processed as above with the addition of Accumax (Stem Cell Technologies) to the final 0.04% bovine serum albumin solution. Guinea pig tissue was processed as with the mouse with the following modifications: a wide-bore 1-ml pipette tip was substituted for the 16-gauge needle, and 0.2 mg ml^−1^ collagenase I (17018029, Thermo Fisher) was added to the digestion solution. For tenrec and guinea pig, a fractionated protocol was adopted to minimize exposure of fragile cells to digestive enzymes. For these species, cells that had dislodged following each passage step were separated from intact tissue and immediately passed through a 70-μm cell strainer into 2 ml of charcoal-stripped fetal bovine serum (100–199, Gemini), while remaining intact tissue was allowed to continue with further digestion. Each fraction was centrifuged at 300*g* for 5 min and resuspended in 500 μl ACK red blood cell lysis buffer (A1049201, Thermo Fisher) for 5 min, then centrifuged at 500*g* for 2 min and resuspended in 0.04% bovine serum albumin (A9647, Sigma) and kept on ice until dissociation was complete. Cells were counted on a haemocytometer and recombined in a proportional manner before sequencing.

Cells were captured using the 10X Chromium platform (3′ chemistry, version 3), and libraries were generated according to manufacturer protocols (CG000315). Libraries were sequenced using an Illumina NovaSeq by the Yale Center for Genomic Analysis (*M. domestica* and *T. ecaudatus*) and at the Next Generation Sequencing Facility of Vienna Biocenter (*C. porcellus* and *M. musculus*).

### Histology and histochemistry

Histological samples were fixed for 24 h in 10% neutral buffered formalin, followed by dehydration to 70% ethanol and kept at 4 °C (days–weeks) or −20 °C (months) before paraffin embedding. Slides for brightfield histology were dewaxed with xylene, rehydrated into water and stained with Gill 2 hematoxylin for 2 min and Y alcoholic eosin for 1 min before being dehydrated in ethanol, xylene treated and mounted in Permount resin. Brightfield images for Fig. 1 were taken with a Leica Thunder Imager (*Tenrec*), a Nikon Eclipse E600 (*Monodelphis*) and an EVOS M7000 (*Cavia* and *Mus*).

Immunohistochemistry was performed using antibodies recorded in Supplementary Table 5. For chromogenic immunohistochemistry, slides were incubated for 1 h at 65 °C, dewaxed with xylene, washed in 100% ethanol and rehydrated in running tap water. Antigen retrieval was performed for 1 h in a 95 °C vegetable steamer containing 10 μM sodium citrate (pH 6.0). Slides were washed in phosphate-buffered saline and blocked with 0.1% mass/volume bovine serum albumin. Peroxidases were blocked by incubation in 0.03% hydrogen peroxide containing sodium azide (DAKO) in a humidification chamber for 30 min. Slides were incubated overnight at 4 °C in primary antibody solutions, blocked and treated with horseradish peroxidase-conjugated secondary antibody for 1 h at room temperature. Finally, slides were washed in blocking solution once more and treated with 3,3′-diaminobenzidine (DAKO, K401011-2) for 5 mins and counterstained with Gill 2 hematoxylin before brightfield imaging.

For fluorescent immunohistochemistry, the procedure had the following modifications: peroxidase blocking and 3,3′-diaminobenzidine was not used in favour of secondary antibodies conjugated with the fluorophore. Images were acquired using an EVOS M7000 Imaging System (Invitrogen). Fluorescent images in the red channel were also captured and superimposed to leverage the autofluorescent nature of erythrocytes to highlight the location of vasculature.

### In situ hybridization

Single-molecule fluorescence in situ hybridization was conducted on formalin-fixed paraffin-embedded sections of 6-μm thickness using the RNAscope platform. Fluorescent RNAscope was performed using the MultiPlex platform (ACD Bio, 323100) and chromogenic RNAscope was performed using the RNAscope 2.5 HD Brown Assay (ACD Bio, 322300). Sequence-specific probe sets were designed against the targeted transcript sequences used for RNA-seq alignment and deposited into the RNAscope database as standardized probes. Probes used are listed in Supplementary Table 6.

All steps followed the manufacturer’s protocols (ACD Bio, 323100-USM for fluorescent and 322310-USM for chromogenic). In brief, after baking for 1 h at 60 °C, slides were deparaffinized, treated with hydrogen peroxide, and target retrieval was performed by submersion in boiling target retrieval solution. Target-specific probes were hybridized alongside slides with bacterial dapB negative control probes and amplification steps were performed in a HybEz oven (ACD Bio). Fluorescent probes were visualized using Opal 520, Opal 570, Opal 620 and Opal 690 fluorophores (Akoya Biosciences). Fluorescent images were captured using a laser scanning confocal microscope (Leica Stellaris 8 Falcon) and brightfield images were captured using an EVOS M7000 microscope. For Fig. 3b, fluorescent images were also captured at 690 nm and superimposed to leverage the autofluorescent nature of the opossum glands to highlight the location of glandular epithelia.

### Single-cell data analysis

Reads were obtained in FASTQ format from the respective sequencing cores. Additional stages of *M. musculus* fetal–maternal interface spanning 11.5–14.5 dpc were downloaded from the NCBI Gene Expression Omnibus (GEO) (GSE156125 (ref. ^31^), GSE196825 (ref. ^80^) and GSE152903 (ref. ^81^)). *M. fascicularis* reads were downloaded from the GEO (GSE180637 (ref. ^8^)). *H. sapiens* aligned counts were downloaded from https://www.reproductivecellatlas.org in pre-aligned form to preserve patient privacy.

Reads were aligned to reference genomes using the 10X Genomics CellRanger software (≥v7.0.0). *Monodelphis domestica*, *Cavia porcellus*, *Mus musculus* and *Macaca fascicularis* were mapped to their respective Ensembl genome annotations (ASM229v1 v104, cavPor3 v104, GRCm39 v110 and Macaca_fascicularis_6.0 v112, respectively). For *Tenrec ecaudatus*, a novel unpublished genome was provided by the laboratory of Michael Hiller and Fauna Bio, annotated using the TOGA pipeline^82^.

Quality control included filtering cells with low transcriptomic complexity (less than 500 unique features for all species except *Monodelphis*) or high mitochondrial gene expression (more than 25%), followed by doublet detection by scrublet (v0.2.3)^83^ and doubletdetection (v4.2)^84^, to remove clusters consisting of majority doublets. Library size normalization, log transformation, feature selection and dimensionality reduction were performed using scanpy ≥v1.9.1^85^. Principal components were adjusted to correct for batch effect across biological replicates using harmony (harmonypy, v0.0.9)^86^. Uniform manifold approximation and projection (UMAP) embeddings were calculated and Leiden^87^ clustering (resolution = 0.8–1.4) was performed to partition cells into putative cell types. Owing to varying degrees of cell heterogeneity, certain cell populations essential to our conclusions (trophoblast and stroma) were sub-clustered by recalculation of principal component analysis (PCA) and UMAP embeddings followed by low-resolution (0.2–0.4) Leiden clustering. Marker genes were calculated based upon differential expression (logistic regression, Wilcoxon rank-sum test and *t*-test as implemented by scanpy.pp.rank_genes_groups) and used to annotate groups. Cells were annotated using a combination of these markers and active genes belonging to NMF gene expression modules (see below), and cell clusters lacking uniquely distinguishable NMF gene modules or markers were merged. Fetal cells were identified by a combination of placenta-specific marker gene expression, and non-overlap with non-pregnant uterine samples.

Select species-specific adjustments to the above process were made. *Monodelphis* libraries had increased numbers of mRNA reads outside valid cells, indicative of background mRNA, probably due to apocrine blebbing observed at this stage (Extended Data Fig. 4). Therefore the following adjustments were made: cellbender (v0.3.0)^88^ was used to infer and remove background mRNA contamination, dimensional reduction and clustering were calculated on cellbender-corrected expression matrices, and a lower cutoff of 200 unique features per cell was used. In downstream analyses including SAMap and communication inference, CellRanger counts were used to ensure methodological consistency. In the case of the tenrec, which was sufficiently outbred, single nucleotide polymorphism-based inference of the genome of origin was conducted with souporcell (v2.5)^89^; Extended Data Fig. 2). Clusters of cells that souporcell assigned to the genome of lesser abundance (putative fetal) overlapped with those annotated as fetal by marker gene expression.

Quality control metrics and details of replicate contributions to annotated clusters are reported in Source Data Extended Data Fig. 2. The sample size of typical single-cell RNA sequencing studies complicates statistical testing of differential cell type abundance^90^, so the relative abundance measures in Fig. 1d are presented as pooled values. Annotated cell type descriptions and marker genes used in annotation are provided in Supplementary Table 1.

### Trajectory analysis

To analyse continuous developmental processes in select cell types, subsets of trophoblastic and endometrial stromal cells were re-embedded in force atlas projections using the scanpy.tl.draw_graph() function. Connectivities between trophoblast cell clusters were calculated using PAGA graph abstraction^91^ via the scanpy.tl.paga() function. Spliced and unspliced transcript calling was performed using the velocyto package (v0.17.17)^92^, and velocity was modelled using scVelo (v0.3.3)^45^ using the stochastic velocity model.

### Homology inference by SAMap

SAMap (v.1.3.4)^13^ was used to project cells from all species in a common manifold and to calculate gene homology-aware similarity scores between cells of different species. BLAST (v2.14.1)^93^ graphs were generated from the ENSEMBL proteomes of respective species, with the exception of *Tenrec ecaudatus*, for which the transcriptome was used with tblastx mode. BLAST graphs were used alongside quality-filtered count matrices to generate a common SAMap embedding for all species using the samap.run() procedure in pairwise mode with otherwise default parameters. The common SAMap embedding showed thorough cross-species mixing, with an overall alignment score^94^ of 0.83 out of 1. Pairwise cell type–cell type mapping scores were calculated using the get_mapping_scores() function on the SAMap graph for all cross-species cell type pairs, and gene–gene pairs driving specific linkages were identified using the find_genes() function.

To more rigorously investigate uncertainty in mapping scores, we used a bootstrapping approach. SAMap mapping scores were re-calculated on 200 random 30% subsamples of the full SAMap graph. The mean scores of all cell type pairs over the 200 iterations and the 95% confidence interval of bootstrapped scores are reported in Supplementary Table 2.

SAMap mapping scores were used to build networks with edge weights corresponding to mapping scores between cell types for select subsets of the total cell samples—trophoblast (Fig. 2) and stromal cells (Fig. 3). Communities were detected within these SAMap networks using the Leiden algorithm (leidenalg, v0.10.2)^87^. Stability of the resulting communities was assessed by repeating Leiden community detection on mapping scores from all 200 iterations from the bootstrapping procedure and calculating a co-occurrence matrix to measure how frequently cell type pairs were assigned to the same cluster across iterations (Extended Data Fig. 5a,b). The stability of the final clusters reported in Fig. 2 and Fig. 3 was defined as the mean of all co-occurrence values between cell type pairs in the final community.

### Gene expression program identification and cross-species comparison

cNMF v1.4.1^11^ was used to infer gene expression programs from count matrices of each species. Analysis was performed following a modified protocol from ref. ^95^. Optimal numbers of factors (*K*) were chosen based on manual examination of stability–error curves over a range of possible *K* values from 10 to 50, selecting a value immediately preceding a sudden drop in stability score (Extended Data Fig. 3).

For cross-species comparison, spectra scores from cNMF (that is, gene loadings in NMF factors) were used. Non-human genes were mapped to their closest human orthologues as determined by BLAST score. In cases of many-to-one mapping, the highest loading score was used. Pearson correlation was calculated between programs, and hierarchical clustering was conducted using the clustermap function in seaborn (v0.13.1).

### In vitro decidualization of primary *Tenrec* stromal cells

Stromal cells were isolated from the mid-gestation *Tenrec ecaudatus* uterus by differential attachment. Cells were expanded in T25 flasks in a growth medium consisting of 15.5 g l^−1^ Dulbecco’s modified Eagle’s medium (DMEM; 30-2002, ATCC), 1.2 g l^−1^ sodium bicarbonate, 10 ml l^−1^ sodium pyruvate (11360, Thermo Fisher) and 1 ml l^−1^ ITS supplement (354350, VWR) in 10% charcoal-stripped fetal bovine serum (100–199, Gemini), and then seeded into 12-well plates (3.9 cm^2^) for 1 day. Samples were treated with either base medium (Base), 1 μM MPA (M1629, Sigma-Aldrich), or 1 μM MPA and 0.5 mM 8-Br-cAMP (B7880, Sigma-Aldrich) for 6 days, with replenishment on day 3 (*n* = 4 for each condition, total *n* = 12). Bulk RNA was isolated using a Qiagen RNeasy Micro Kit (74004), libraries were prepared by the Yale Center for Genomic Analysis and sequenced using an Illumina NovaSeq. Reads were aligned to the draft genome using kallisto (v0.48.0). Significance of differentially expressed genes was calculated using the Wald test function of pyDESeq2 (v0.4.10)^96^ with adjustment for multiple testing using the Benjamini–Hochberg method.

### Cell–cell communication analysis

A ground truth ligand–receptor database was built as a manually extended fork of CellPhoneDB v5.0.0^97^ with additional curation and metadata. This modified list is available at https://gitlab.com/dnjst/ViennaCPDB/ and archived via Zenodo^98^. Because the ground truth database was curated from the human literature and insufficient data exist to curate ligand–receptor pairs for each species, non-human transcriptomes were converted into ‘human-equivalent transcriptomes’ by mapping of genes to their top human orthologue as detected by BLAST of translated peptide sequence^13^. To maximize coverage, in cases of many:one human orthology, counts of all detected paralogues were pooled together. This transformed expression matrix was used only for cell communication analysis: dimensionality reduction, cluster identification, differential expression testing, and marker gene identification and plotting were conducted on unadulterated species-relevant gene sets.

From the ground truth list, interaction scores for all cell type pairs in our transcriptomes were generated using expression thresholding with a cutoff of 20% of cells in a cluster for ‘on’ (https://gitlab.com/dnjst/chinpy; v0.0.55), followed by statistical testing for significantly cell-type-enriched interactions using the LIANA+ (v1.5.0)^99^. Single-cell transcriptomes from multiple biological replicates (sample sizes reported above) were pooled before inference of cell communication was performed, following common practice^100^. Rather than biological replicate-based statistical testing, statistical tests of signalling specificity designed for single-cell data were called via the liana.method.rank_aggregate() function, which executes permutation tests from CellPhoneDB^101^ and CellChat^102^.

For co-evolutionary integration analysis, all ligand–receptor pairs in the ground truth database were evaluated as either ‘off’ (ligand and receptor off), ‘allocrine ligand’ (ligand on, receptor off), ‘allocrine receptor’ (receptor on, ligand off) or ‘autocrine’ (ligand and receptor on) states for all cells in the sample, with ‘on’ defined as non-zero expression. Classification of ligands was done in this manner to measure intrinsic signalling potential for each individual cell in a way that is not inflated by the number of clusters of other cell types identified in the tissue, in contrast to network analysis, where the splitting of a given cell type into multiple clusters inflates the number of inferred outgoing interactions for other cell types.

We define the ALC of a cell *c* as:

$${{\rm{AL}{C}}}_{c}=\mathop{\sum }\limits_{i=1}^{N}\qquad{L}_{i,c}(1-{R}_{j(i),c})$$

where *L*_*i*,*c*_ and *R*_*j*(*i*),*c*_ represent the binarized expression statuses of ligand *i* and its corresponding receptor *j*(*i*) in cell *c*. Expression status is defined such that *L*_*i*,*c*_ = 1 if ligand *i* is captured in cell *c* with at least 1 count, otherwise 0, and *R*_*j*(*i*),*c*_ is defined likewise for receptor *j*(*i*)). The sum is taken over *N* ligand–receptor pairs in the curated database from above. Statistical associations between ALCs and cell type class of the cluster (for example, lymphatic) were assessed using one-way ANOVA performed on average ALC scores for each unique species–cell cluster–biological replicate combination (for example, Mm_TC_replicate1 for mouse T cells in batch 1).

To analyse graph statistics at the level of cell types, multi-edge graphs were constructed with nodes equal to cell types and edges for each potential ligand–receptor interaction with weights set to the product of ligand and receptor log-normalized transcripts per 10,000, a metric used to quantify signalling magnitude^103^. Kleinberg hub and authority scores^57^ were calculated on these graphs for all cell types within their respective species signalling networks using the HITS algorithm as implemented in the hub_score() and authority_score() functions in igraph (v0.11.6).

For disambiguation analysis, ligands from the ground truth database were grouped into ligand families using annotations modified from the CellPhoneDB ‘classification’ metadata category to correct omissions and ensure each ligand belonged to only one family (archived at https://gitlab.com/dnjst/fmi2024). In cases of many-to-one homology, all possible homologues to the human ground truth ligand list were added as separate potential ligands, based on Ensembl (mouse, guinea pig, opossum) or BLAST (tenrec) orthology to the human.

### Ancestral state reconstruction

Ancestral state reconstruction was performed using cell type-level pseudo-bulk transcriptomes for which both per cent abundance and expression magnitude levels were calculated for all genes encoding ligand or receptor components present in the ground truth interactions database. These were then aggregated in the following ways to assign comparable species-level character states.

For discrete ancestral state reconstruction, genes were scored in a binary off/on manner based on a threshold of 20% of cells in a cluster having non-zero expression. To reduce complexity of cross-species cell type homology, these values were collapsed by cell type class (epithelial, trophoblastic, myeloid and so on) by a criterion that at least one cell type passes the 20% threshold: for example, if extravillous trophoblast expressed IGF1 past the 20% threshold, the state for the character ‘Trophoblastic|IGF1’ was set to 1.

Four discrete ancestral state reconstruction models were compared. Wagner parsimony was calculated using the MPR() function in the ape package (v5.8)^104^ with *M. domestica* as the outgroup. Maximum parsimony was calculated using the asr_max_parsimony() function of the castor package (v1.8.0)^105^ with two transition models: first, a transition matrix weighting expression gains as twice the cost of losses, parameters found to be biologically plausible for gene gain and loss^106,107^; and second, a simple maximum parsimony model with equal weights assigned to gains and losses. Finally, restricted maximum likelihood on expression proportion values (0–1 proportions of cells with non-zero expression in the leading cluster of the pool under consideration) was calculated via the ace() function in the ape package with type = ‘continuous’ and method = ‘REML’ arguments, from which states were binarized based on a 20% cutoff. The majority (15,634/18,875, 83%) of character state configurations inferred for the five internal nodes in the tree were identical between the four methods (Extended Data Fig. 8), with most differences attributable to whether gains on the *Monodelphis* branch are allowed (disallowed in the Wagner model). States reported in Fig. 4 derive from the equal weights maximum parsimony model.

Gene expression gains were calculated for all internal branches of the six-species tree by identifying genes considered ‘on’ in descendant nodes but inferred ‘off’ in the immediate common ancestor node, or vice versa for losses. To reconstruct cell–cell interaction networks in common ancestral species, the reconstructed ancestral binary gene presence/absence matrices were used to infer possible signalling interactions between cell type classes by the requirement that all ligand subunits must be present in the sending cell and all receptor subunits must be present in the receiving cell, for all possible cell–cell pairs tested.

For continuous ancestral state reconstruction used in the escalation analysis (Fig. 5e,f and Extended Data Fig. 8f), cell type resolution (pseudo-bulk) expression values for all genes were calculated in transcript counts per million (TPM) scale. Only genes with non-zero expression in at least four of six species were retained (that is, up to two losses of expression or locus were tolerated). In order to trace the evolution of individual signalling channels despite events of gene expression redistribution among (such as swapping of ligand production between trophoblast cell types) and reduce dependency of results upon precise cell type homology, the expression levels of the fetal and maternal cell types with the highest expression of ligand and receptor, respectively, were chosen as representative trait values for each species. The biological interpretation of this approach is that the cell types with greatest ligand and receptor gene expression in the fetus and mother are those most likely to be co-evolving with respect to the signalling pair in question. To mitigate bias due to differences in dynamic range of gene expression between species, TPM values were standardized using a zFPKM-style transformation^108^ as implemented in R (zFPKM v1.28.0). This approach fits a Gaussian distribution with mean *μ* and standard deviation *σ* to log_2_-transformed expression values within each species then calculates standardized *z*-scores (zTPM) according to the following formula:

$${{\rm{zTP}{M}}}_{i}=\frac{{{\rm{lo}{g}}}_{2}({{\rm{TP}{M}}}_{i})-\mu }{\sigma }$$

That is, if extravillous trophoblast expressed IGF1 at a zTPM level of 2.5, the state for the character ‘Fetal|IGF1’ would be set to 2.5. A zTPM lower limit of −3, based on the original paper’s defined lower bound for biologically relevant gene expression^108^, was imposed. From these values, restricted maximum likelihood was used to estimate ancestral states as above. Changes in continuous expression values for ligands and receptors along internal branches of the six-species tree were calculated by subtracting descendant node expression values from inferred immediate ancestral node values. For the purposes of plotting growth factors separately in Fig. 5f and Extended Data Fig. 8, growth factors were defined as ligands belonging to Gene Ontology group GO:0008083.

### Statistical analysis of escalation

For escalation analysis, we tested whether ligand and receptor expression co-evolve using phylogenetic independent contrasts (PICs)^62^ to account for shared evolutionary history. A reduced copy of the forked CellPhoneDB ligand–receptor database was used to generate the candidate testing pairs by cutting the interactions to only secreted ligands and their cognate ligand-binding subunit genes, excluding co-receptors. PICs were calculated for ligand and corresponding receptor expression values (in zTPM scale as above) using the pic() function in ape (v5.8)^104^ and a time-calibrated phylogeny of our six species from TimeTree 5^109^. Linear regression was performed on the PIC values using the model design ‘ligand PIC ~ receptor PIC + 0’. That is, with ligand PIC as the independent variable and receptor PIC as the dependent variable, and the intercept forced to zero (assuming that when there is no change in ligand expression, there is no change expected in receptor expression). Linear regression was performed on observed expression values to return a regression slope between ligands and receptors across species. *P* values reported as ‘PIC *P* value’ in Fig. 5e result from a two-sided *t*-test on the estimated slope coefficient of linear regression through the origin of ligand versus receptor PICs. *P* values are reported in unadjusted form, after correction for multiple testing using the Bonferroni correction, as well as after the Benjamini–Hochberg procedure using the base R function p.adjust(method = ‘BH’) (Source Data Fig. 5). Unadjusted *P* values are plotted on the vertical axis in Fig. 5e with a Bonferroni-adjusted cutoff plotted for visualization. In parallel, standard ordinary least squares regression slopes were calculated for each ligand–receptor pair using raw (non-contrast) ligand expression values with the model ‘ligand zTPM ~ receptor zTPM’ and are plotted on the horizontal axis in Fig. 5e. These slopes reflect the magnitude and direction of association across species before phylogenetic correction.

### Statistical analysis of disambiguation

The disambiguation hypothesis was tested by comparing the observed co-expression status of ligands to the expected exclusively maternal, exclusively fetal and co-expressed ligands under a null model where ligand expression is randomly redistributed. All secreted peptide and small-molecule-mediated ligand–receptor interactions were classified into ‘fetal-only’, ‘maternal-only’ or ‘both’ expression groups based on the threshold value of 20% or more of the cells sequenced expressing the ligand. Ligands were grouped into ligand families using a curated version of the CellPhoneDB (v5.0.0) ‘classification’ metadata. For each ligand family, the per-cell type probability of a given ligand being expressed under the null model was calculated according to the equation:

$${P}_{{{\rm{on}}}}=\frac{{L}_{{{\rm{expressed}}}}}{{S}_{{{\rm{pathway}}}}\times ({N}_{{{\rm{fetal}}}}+{N}_{{{\rm{maternal}}}})}$$

where *L*_expressed_ is the observed number of ‘on’ calls within the ligand family across fetal and maternal cell types, *N*_fetal_ is the number of fetal cell types, *N*_maternal_ the number of maternal cell types and *S*_pathway_ is the number of ligands in the family.

The null probabilities of fetal-only and maternal-only expression for a ligand, respectively, were given by:

$${P}_{{{\rm{fetal}}\;{\rm{only}}}}=\left(1-{(1-{P}_{{{\rm{on}}}})}^{{N}_{{{\rm{fetal}}}}}\right)\times {(1-{P}_{{{\rm{on}}}})}^{{N}_{{{\rm{maternal}}}}}$$

$${P}_{{{\rm{maternal}}\;{\rm{only}}}}=\left(1-{(1-{P}_{{{\rm{on}}}})}^{{N}_{{{\rm{maternal}}}}}\right)\times {(1-{P}_{{{\rm{on}}}})}^{{N}_{{{\rm{fetal}}}}}$$

The expected fetal-only and maternal-only ligands in each family were obtained by multiplying *P*_fetal only_ and *P*_maternal only_ by the number of ligands in the family.

Finally, a one-way chi-squared goodness-of-fit test for identity of observed and expected counts of the three scoring categories (co-expressed, fetal-only and maternal-only) was used to obtain chi-squared and *P* values, with individual observations corresponding to ligands within a family. The Benjamini–Hochberg correction was used (statsmodels.multitest.multipletests v0.14.1) with an alpha of 0.05 to obtain false discovery rate *q* values. For plotting, ligand families were considered significant if *P* ≤ 0.05 and *q* ≤ 0.05. Bonferroni-corrected *P* values, obtained by multiplying *P* by the number of ligands in a family, are reported in Source Data Fig. 5.

### Statistics and reproducibility

The numbers of replicate sequencing libraries analysed were as follow: *M. domestica*: 2; *T. ecaudatus*: 3; *C. porcellus*: 2; *M. musculus*: 13; *M. fascicularis*: 23; *H. sapiens*: 62.

All immunohistochemical and in situ hybridization experiments were performed alongside no-primary-antibody/bacterial dapB in situ probe negative controls, respectively, on paired tissue sections. For immunohistochemical experiments, tissues were stained in triplicate at a series of primary antibody concentrations as listed in Supplementary Table 5. Representative images are shown. For RNAscope in situ hybridization, only a single concentration was used, per manufacturer specifications.

### Reporting summary

Further information on research design is available in the Nature Portfolio Reporting Summary linked to this article.

## Supplementary information

Reporting Summary

Supplementary Tables Details on abbreviations used and hierarchical classification of cell type clusters in all species. Output from cross-species mapping (SAMap) with full pairwise mapping scores plus 95% confidence intervals, and top cross-species gene–gene pairs driving trophoblast and stromal cell type linkages.

## Source data

Source Data Fig. 3 NMF weights of genes identified by cNMF as the top-expressed gene expression program in stromal cell types across species.

Source Data Fig. 4 Character states for ancestral state reconstruction of ligand–receptor signalling.

Source Data Fig. 5 Co-expression state calls from disambiguation analysis, disambiguation hypothesis testing statistics, escalation hypothesis testing statistics (phylogenetic independent contrasts).

Source Data Extended Data Fig./Table 2 Spreadsheet with quality control information on scRNA-seq libraries, contribution of technical replicates to cell type cluster composition.

Source Data Extended Data Fig./Table 8 Ligand and receptor branch changes for testing of the escalation hypothesis.

## Data Availability

New sequencing data have been uploaded to the NCBI Gene Expression Omnibus (GEO) at accession number GSE274701. Human gene expression data used in this analysis were retrieved from https://www.reproductivecellatlas.org/ in aligned form to preserve patient privacy, macaque data were retrieved from accession number GSE180637, and additional mouse data were retrieved from accession numbers GSE15612531, GSE19682579 and GSE152903. No unique materials in the form of cell lines were used in the study, and cells were used for destructive sampling. Remaining histological material is stored at the University of Vienna and available for research upon request. Source data are provided with this paper.

## Code availability

Analytical code for this study is available at https://gitlab.com/dnjst/fmi2024 and via Zenodo at 10.5281/zenodo.15337652 (ref. ^98^). Additional processing scripts for cell–cell communication data are available in a python package at https://gitlab.com/dnjst/chinpy.
